# Liraglutide to Improve corONary haemodynamics during Exercise streSS (LIONESS): a double-blind randomised placebo-controlled crossover trial

**DOI:** 10.1186/s13098-021-00635-6

**Published:** 2021-02-12

**Authors:** Aung Myat, Simon R. Redwood, Satpal Arri, Bernard J. Gersh, Deepak L. Bhatt, Michael S. Marber

**Affiliations:** 1 King’s College London British Heart Foundation Centre of Research Excellence, The Rayne Institute, Cardiovascular Division, St Thomas’ Hospital, Westminster Bridge Road, London, SE1 7EH UK; 2 Frimley Park Hospital, Frimley Health NHS Foundation Trust, Portsmouth Road, Frimley, GU16 7UJ Camberley UK; 3 Department of Cardiovascular Medicine, Mayo Clinic College of Medicine, 200 First Street SW, Rochester, MN 55905 USA; 4 Brigham and Women’s Hospital Heart & Vascular Centre and Harvard Medical School, Boston, MA 02115 USA

**Keywords:** Liraglutide, Glucagon-like peptide-1 receptor agonist, Myocardial ischaemia, Chronic stable angina, Coronary artery disease, Incretin

## Abstract

**Background:**

Glucagon-like peptide-1 receptor (GLP-1R) activation may improve myocardial performance in the context of ischaemia, independent of glycaemic control, in individuals with and without type 2 diabetes mellitus.

**Methods:**

The LIONESS trial was a single-centre randomised double-blind placebo-controlled crossover study to determine whether prolonged GLP-1R activation could improve exercise haemodynamics in chronic stable angina patients. Eligibility criteria comprised angiographic evidence of obstructive coronary artery disease (CAD) and an abnormal baseline exercise tolerance test (ETT) demonstrating > 0.1 mV of planar or downsloping ST-segment depression (STD). Those randomised to active agent started with a 1-week run-in phase of 0.6 mg liraglutide daily, an established injectable GLP-1R agonist, followed by 1 week of 1.2 mg liraglutide, after which patients performed a week 2 ETT. Patients then self-administered 1.8 mg liraglutide for a week before completing a week 3 ETT. The placebo arm received visually and temporally matched daily saline injections. Participants then crossed over to a 3-week course of saline injections interspersed with a week 5 ETT and week 6 ETT and vice versa. Co-primary endpoints were rate pressure product (RPP) at 0.1 mV STD and magnitude of STD at peak exercise.

**Results:**

Twenty-two patients (21 without diabetes) were randomised. There was no significant difference between saline versus liraglutide in the co-primary endpoints of RPP achieved at 0.1 mV STD (saline vs. liraglutide 1.2 mg p = 0.097; saline vs. liraglutide 1.8 mg p = 0.48) or the degree of STD at peak exercise (saline vs. liraglutide 1.2 mg p = 0.68; saline vs. liraglutide 1.8 mg p = 0.57). Liraglutide did not cause symptomatic hypoglycaemia, renal dysfunction, acute pancreatitis or provoke early withdrawal from the trial. Liraglutide significantly reduced weight (baseline 88.75 ± 16.5 kg vs. after liraglutide 87.78 ± 16.9 kg; p = 0.0008) and improved the lipid profile (mean total cholesterol: at baseline 3.97 ± 0.88 vs. after liraglutide 3.56 ± 0.71 mmol/L; p < 0.0001).

**Conclusion:**

Liraglutide did not enhance exercise tolerance or haemodynamics compared with saline placebo during serial treadmill testing in patients with established obstructive CAD. It did, however, significantly reduce weight and improve the lipid profile.

*Trial Registration* ClinicalTrials.gov Identifier NCT02315001. Retrospectively registered on 11th December 2014.

## Background

Cardiovascular morbidity and mortality predominates in diabetes with at least a two-fold excess risk of developing ischaemic heart disease, stroke, peripheral arterial disease and heart failure [1]. Efforts to ameliorate this risk by intensifying glycaemic control have, for the most part, had limited success [2–5] and in the case of rosiglitazone, an association with increased adverse cardiovascular events [6]. Regulatory guidance concerning the safety of novel antidiabetic agents followed, mandating the need for large pre- and post-approval outcomes trials in type 2 diabetes mellitus (T2DM) patients already at high cardiovascular risk. Several of these trials have studied incretin-based antidiabetic therapies, namely dipeptidyl peptidase-4 (DPP-4) inhibitors and glucagon-like peptide-1 receptor agonists (GLP-1Ra) [7–14]. These trials were primarily designed to establish cardiovascular safety. They were not conducted as glycaemia lowering intensification studies to demonstrate cardiovascular risk reduction. Nevertheless, the LEADER (liraglutide) and SUSTAIN-6 (semaglutide) trials, in particular, have both demonstrated significant reductions in the primary composite endpoint of cardiovascular death, nonfatal myocardial infarction (MI) and stroke when a GLP-1Ra was added to standard therapy for T2DM versus placebo [10, 15]. Furthermore several preclinical and human studies of GLP-1Ra have shown evidence of direct cardioprotection in the failing heart or the myocardium under threat of ischaemia/reperfusion injury [16–18]. A definitive mechanism by which direct or indirect cardioprotection is mediated remains elusive. There are currently, however, no studies in the literature looking at the potential role of GLP-1Ra in an anti-anginal capacity. This is an important prelude to the use of these agents in the setting of acute MI, since their use could potentially deliver benefits to those who fall victim to silent MI, late presentation or early death before access to medical services. It is in this context that we set out to determine whether the putative anti-ischaemic properties of GLP-1 could translate into an anti-anginal action during sequential exercise stress testing and in so doing demonstrate whether chronic GLP-1 receptor activation could reproduce the beneficial sequelae of the warm-up angina effect without the patient first having to perform symptom-limiting, ischaemia-provoking exercise.

## Methods

The Liraglutide to Improve coROnary haemodynamics during Exercise streSS (LIONESS) trial (NCT02315001) was an investigator-initiated single-centre randomised double-blind placebo-controlled crossover proof-of-principle study designed to characterise the physiological effect of chronic GLP-1R activation on validated haemodynamic and electrophysiological parameters during serial exercise treadmill testing (ETT) in patients shown to have inducible myocardial ischaemia and obstructive coronary artery disease (CAD). The hypothesis being chronic GLP-1R activation can positively augment exercise haemodynamics during serial exercise stress testing when compared with saline placebo. The LIONESS trial was conducted in accordance with the principles of the Declaration of Helsinki and Good Clinical Practice. All participants provided written informed consent prior to randomisation.

### Study population

The full inclusion and exclusion criteria can be found in Additional file 1. Participants were required to have chronic stable ischaemic heart disease (SIHD), either managed conservatively, or awaiting elective coronary revascularisation either by percutaneous coronary intervention (PCI) or coronary artery bypass graft (CABG) surgery. Eligible individuals had angiographic evidence of a > 70% stenosis in a main epicardial coronary artery, with or without coronary stenoses elsewhere, a normal resting electrocardiogram (ECG) in sinus rhythm without bundle branch aberration or other conduction disturbance and a recent/baseline abnormal ETT demonstrating > 0.1 mV of planar or down-sloping ST-segment depression.

### Protocol

The full protocol has been published previously [19]. In brief enrolled participants were randomised 1:1 to Group A (Treatment A then Treatment B sequence) or Group B (Treatment B then Treatment A sequence); i.e. starting with liraglutide or matched-volume saline placebo.

Participants had their heart rate-limiting drugs cautiously withdrawn prior to study commencement. Long-acting oral nitrates and nicorandil were also discontinued to avoid potential interference with haemodynamics and masking of angina burden (Additional file 1: Table S1). After trial completion individuals were recommenced on all baseline pharmacotherapy.

**Fig. 1:**
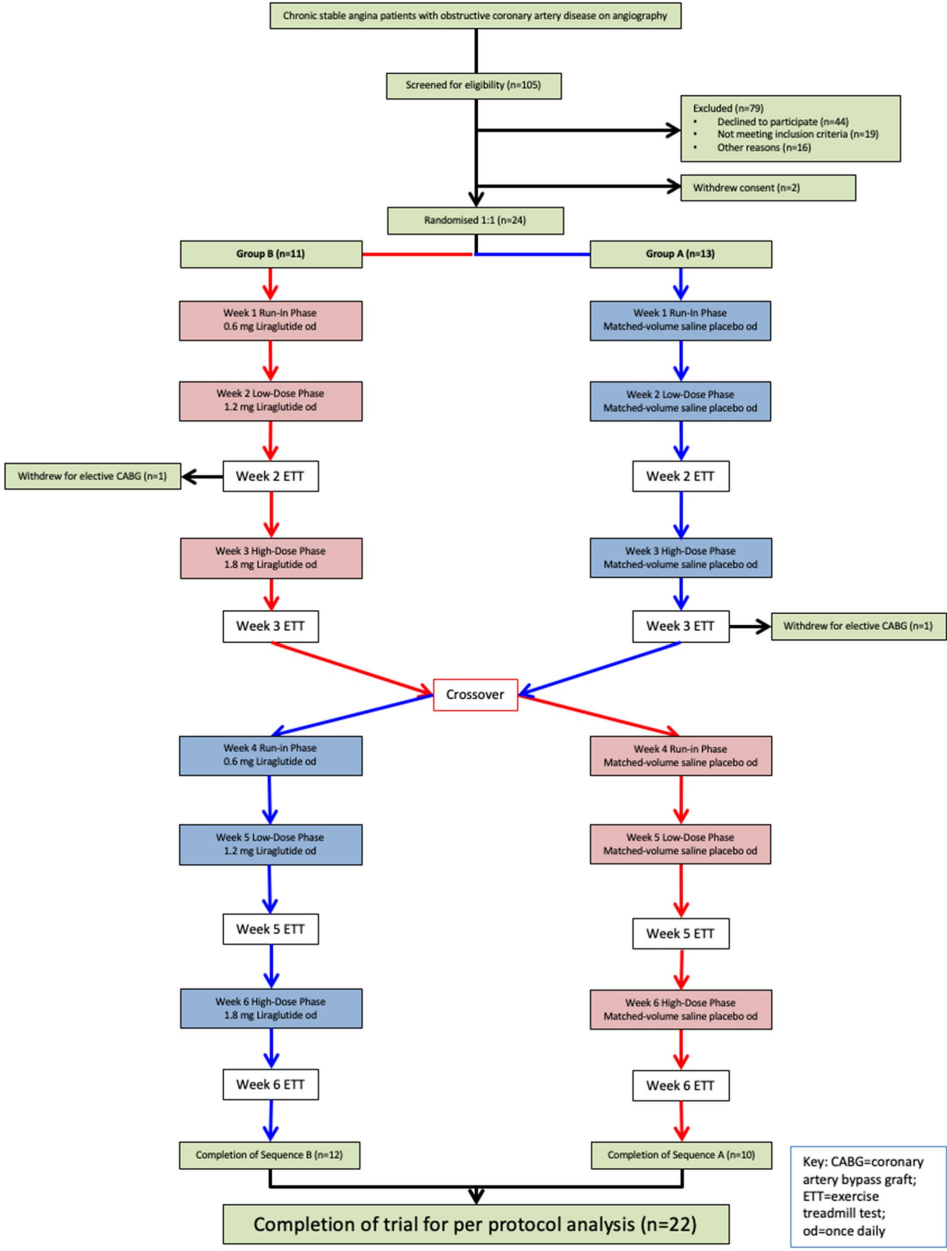
The LIONESS trial CONSORT diagram

After a 1-week run-in phase of daily 0.6 mg liraglutide followed by a 1-week course of daily 1.2 mg liraglutide, patients in the active intervention arm proceeded to a Week 2 ETT. A run-in phase of once daily 0.6 mg liraglutide was incorporated to encourage tolerance to the known gastrointestinal side effects of GLP-1Ra’s and therefore minimise premature patient withdrawal. Patients were then up-titrated to high-dose 1.8 mg liraglutide for another week before performing a Week 3 ETT. Those patients randomised to the placebo arm were given visually and temporally matched-volume saline subcutaneous injections for the first two weeks before the Week 2 ETT and then another week of saline injections before the Week 3 ETT. Patients then crossed over to complete the 6-week protocol so that those first randomised to the active agent arm switched to the placebo arm and vice versa (Fig. 1). This was a proof of principle trial with no planned follow up beyond the 6-week protocol.

Liraglutide (Victoza®, NovoNordisk, Bagsvaerd, Denmark) is a guideline-approved drug that shares 97% structural homology with native GLP-1. Liraglutide is an appropriate surrogate for studying chronic GLP-1R activation in humans. Furthermore it has a half-life of approximately 10–14 h after subcutaneous injection, which allows for use as a once-daily preparation. This permitted a crossover trial design, helped to maximise patient compliance and precluded the risk of fluid overload in individuals with established CAD. The small risk of hypoglycaemia associated with liraglutide lent further support to it being used in an all comers setting.

### Endpoints

#### Co-primary endpoints

- Change in rate pressure product (RPP) at 0.1 mV ST-segment depression (STD)
- Change in magnitude of STD at peak exercise during sequential ETTs

The RPP is the product of heart rate and systolic blood pressure expressed as beats/minute mmHg. It is a recognised indicator of myocardial oxygen consumption (MVO_2_) and a validated marker of cardiac function and overall physical fitness [20]. The RPP can be used to gauge haemodynamic response to exercise and can be used to titrate exercise intensity to remain below the angina threshold in those with chronic stable angina.

#### Secondary inducible ischaemia endpoints

- Change in time to 0.1 mV STD
- Change in time to maximum STD

#### Secondary exercise capacity endpoints

- Change in total exercise time
- Change in recovery time to 0.05 mV STD

#### Secondary safety endpoints

- Evidence of hypoglycaemia
- Evidence of renal dysfunction
- Evidence of acute pancreatitis

All exercise tests were analysed by two independent investigators blinded to treatment allocation and sequence (see Additional file 1). All exercise tests were analysed and the results documented before the trial was un-blinded. A stepwise increase in liraglutide dosage was factored into the trial protocol with the aim of observing a dose–response effect on haemodynamics during serial exercise testing. The ETTs performed after each week during the liraglutide treatment period were analysed independently and compared with the corresponding ETT performed on saline placebo. Therefore the week 2 ETT-1 was compared with the week 5 ETT-3 and the week 3 ETT-2 with the week 6 ETT-4 (Fig. 1).

### Statistical analysis

Previous studies have shown that the time taken to 0.1 mV STD (368 ± 34 → 418 ± 36 s) and the rate pressure product at 0.1 mV STD (20,500 ± 755 → 21,907 ± 764 mmHg/min) are significantly increased during the second of two serial exercise tests separated by 15 min in chronic CAD patients with known left anterior descending artery stenoses [21, 22]. This warm-up angina effect is thought to augment the innate resistance of the myocardium to an ischaemic insult [23, 24]. We postulated that the administration of liraglutide would mimic the beneficial cardioprotective effects of warm-up angina. A sample size of 26 patients randomised in a 1:1 fashion to each treatment arm (taking into consideration a > 10% drop-out rate) followed by crossover would have 90% power to detect a difference between the means of approximately 15% (2-sided α = 0.05).

The LIONESS trial is effectively a two-period two-treatment crossover trial with a basic AB/BA design. The 2-week transition period from the Week 3 ETT to the Week 5 ETT was deemed sufficient to allow adequate washout of the active drug and therefore minimise the impact of a potential carryover effect. The D’Agostino-Pearson test for normality was performed on each of the sample population parameters under investigation followed by analysis of the two sequence groups A-B and B-A. A rule out test for a carryover effect was performed using a parametric unpaired t-test for a normal distribution or a non-parametric Mann Whitney test for a non-normal distribution. If the test yielded a statistically significant result, this would indicate a significant carryover effect from one treatment sequence to the next. In such cases the first period results before crossover were analysed in isolation [25].

In the absence of a carryover effect outcome after Treatment A is compared directly with outcome after Treatment B (i.e. we ignore whether Treatment A is given in Period 1 or Period 2, given that a significant carryover effect would have been ruled out). A parametric paired t-test or a non-parametric Wilcoxon matched-pairs signed rank test was adopted for this analysis, guided by the baseline test for normality. A one-way ANOVA was used for multiple comparisons. Linear regression would be used thereafter to study the correlation between outcome parameters and baseline patient characteristics if a significant treatment effect were to be discovered. The trial was analysed on a per protocol basis. A p value < 0.05 was considered statistically significant. All analyses were conducted using GraphPad Prism (San Diego, CA, USA).

## Results

From December 2013 to October 2014, 105 chronic SIHD patients were screened. Of these, 26 individuals fulfilled the inclusion criteria and consented to participate. Two patients subsequently withdrew consent prior to randomisation and a further 2 withdrew during the trial protocol to proceed to elective CABG surgery. Therefore 22 patients completed the 6-week protocol which met the target sample size. Baseline demographics are shown in Table 1. The mean age (standard deviation—SD) of the per protocol cohort was 62.5 (8.6) years and the mean (SD) body mass index was 29.8 (4.0). Subjects were predominantly male (n = 20/22; 90.9%) and white British (n = 20/22; 90.9%). Most were awaiting elective CABG surgery (n = 20) and the remainder were being managed conservatively (n = 5) or awaiting elective PCI (n = 1). All but two had angiographic multivessel obstructive CAD.

**Table 1:** Baseline demographics of trial participants

	Group A (n = 12)^a^ Saline then liraglutide	Group B (n = 10)^a^ Liraglutide then saline
Mean age ± SD	65.3 ± 7.3	59.2 ± 9.1
Sex	11 male (91.7%)	9 male (90%)
Mean body mass index ± SD	30.1 ± 3.4	29.6 ± 4.8
Severity of CAD	1VD = 2/12 (16.7%) MVD = 10/12 (83.3%)	1VD = 0/12 (0%) MVD = 10/10 (100%)
Prior CVA/TIA	2/12 (16.7%)	1/10 (10%)
Prior MI	4/12 (33.3%)	2/10 (20%)
Prior PCI	2/12 (16.7%)	3/10 (30%)
Prior CABG	1/12 (8.3%)	0/10 (0%)
Hypertension	7/12 (58.3%)	6/10 (60%)
Type 2 diabetes mellitus	1/12 (8.3%)	0/10 (0%)
Hypercholesterolaemia	4/12 (33.3%)	6/10 (60%)
Smoker (past and present)	4/12 (33.3%)	10/10 (100%)
Family history of premature CAD	4/12 (33.3%)	2/10 (20%)

*CABG* coronary artery bypass graft, *CAD* coronary artery disease, *CVA* cerebrovascular accident, *MI* myocardial infarction, *MVD* multivessel disease, *PCI* percutaneous coronary intervention, *SD* standard deviation, *TIA* transient ischaemic attack

^a^Individuals acted as their own controls within the crossover nature of the study protocol. As such no inter-group comparisons have been made

No patient withdrew from the trial prematurely due to adverse events. Furthermore there were no specific adverse events associated with the cautious withdrawal of beta-blockers in the 20 out of 22 individuals that completed the trial protocol. Once the final patient had completed their 6-week protocol, and all exercise tests had been analysed, subsequent un-blinding revealed the treatment sequences (Fig. 1).

### Percentage target heart rate (THR) achieved

**Fig. 2:**
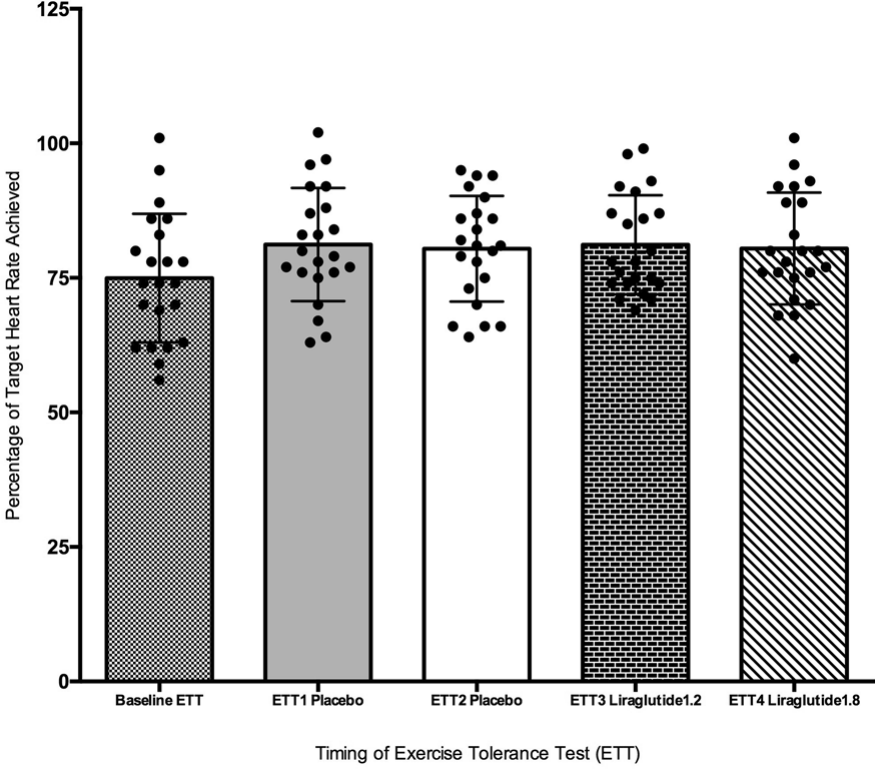
Mean percentage of target heart rate achieved across sequential exercise testing

Mean percentage of THR achieved across all ETTs performed during the trial was approximately 81 ± 10%. Percentage THR achieved by all trial participants at baseline and after each week of treatment (irrespective of treatment period) can be seen in Additional file 1: Table S2. Using a one-way ANOVA with multiple comparisons, there was no significant difference in percentage of THR achieved by all trial participants during all four of the serial ETTs as per protocol. There was, however, a significant difference between the percentage of THR achieved at baseline in comparison to rates achieved during the trial protocol (p = 0.011) (Fig. 2).

### Primary endpoints

#### Change in RPP at 0.1 mV STD

**Fig. 3:**
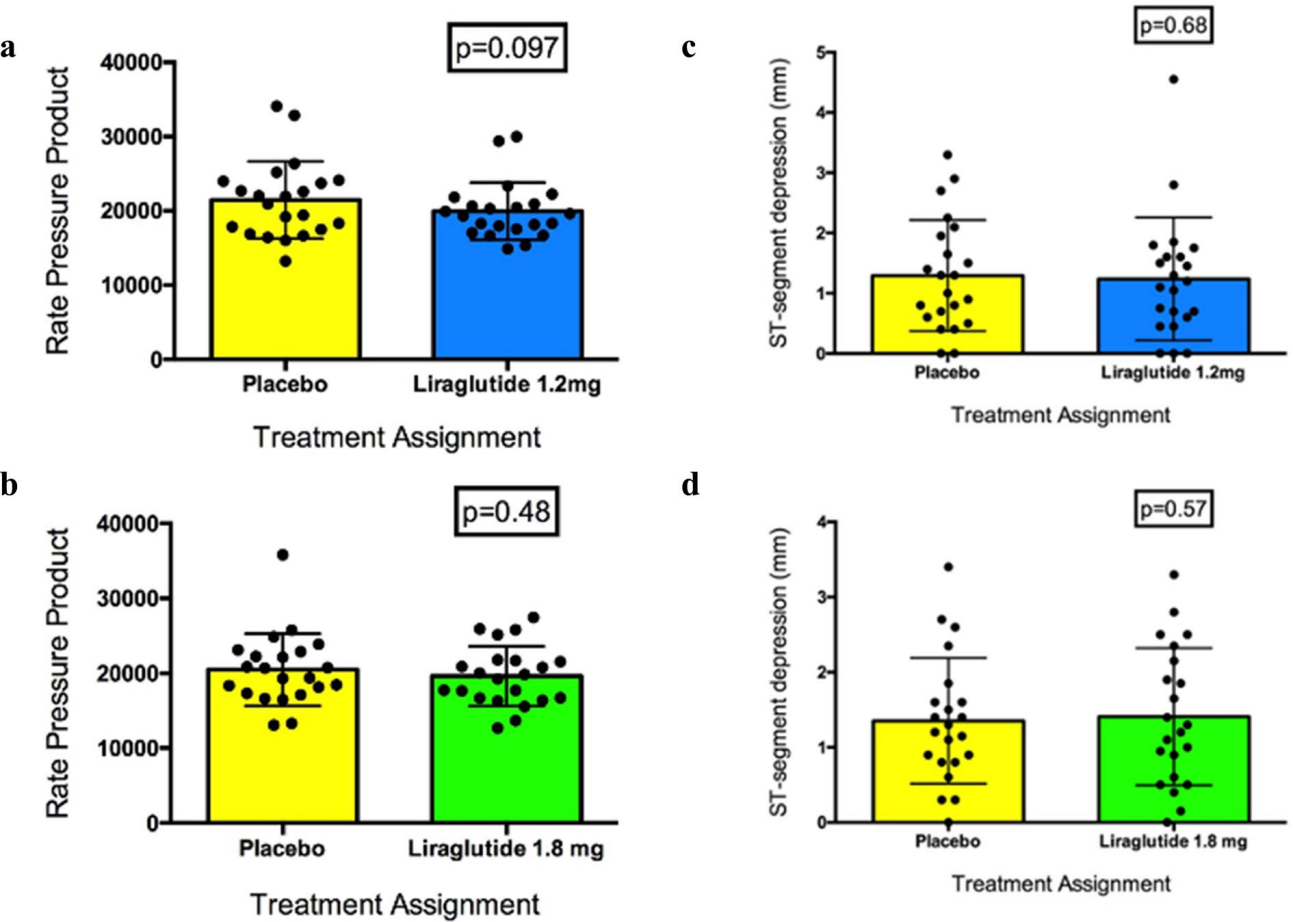
Primary endpoints. **a** Rate pressure product at 0.1 mV ST-segment depression ETT-1 placebo vs. ETT-3 1.2 mg liraglutide. **b** Rate pressure product at 0.1 mV ST-segment depression ETT-2 placebo vs. ETT-4 1.8 mg liraglutide. **c** ST-segment depression at peak exercise ETT-1 placebo vs. ETT-3 1.2 mg liraglutide. **d** ST-segment depression at peak exercise ETT-2 placebo vs. ETT-4 1.8 mg liraglutide

There was no carryover effect during the transition from ETT-1 (matched placebo or liraglutide 1.2 mg) to ETT-3 (1.2 mg liraglutide or matched placebo) (Mann Whitney test p = 0.18) (Additional file 1: Table S3). The test for normality revealed a non-parametric distribution. When the difference between the RPP at 0.1 mV STD after the first ETT on placebo was compared with the RPP at 0.1 mV STD after liraglutide 1.2 mg (i.e. we ignore whether placebo or liraglutide 1.2 mg is given in Period 1 or Period 2), there was no significant treatment effect (Wilcoxon matched-pairs signed rank test p = 0.097) (Fig. 3a).

There was no carryover effect during the transition from ETT-2 (matched placebo or liraglutide 1.8 mg) to ETT-4 (1.8 mg liraglutide or matched placebo) (Mann Whitney test p = 0.381) (Additional file 1: Table S4). The test for normality revealed a non-parametric distribution. When the difference between the RPP at 0.1 mV STD after the second ETT on placebo was compared directly with the RPP at 0.1 mV STD after liraglutide 1.8 mg (irrespective of trial period), no significant treatment effect was discovered (Wilcoxon matched-pairs signed rank test p = 0.483) (Fig. 3b).

#### Change in degree of STD at peak exercise

There was no carryover effect during the transition from ETT-1 to ETT-3 (Mann Whitney test p = 0.76) (Additional file 1: Table S5). No significant treatment effect was seen after comparing the STD at peak exercise between the first ETT on placebo with the ETT on liraglutide 1.2 mg irrespective of trial period (Wilcoxon matched-pairs signed rank test p = 0.684) (Fig. 3c).

There was no carryover effect from ETT-2 to ETT-4 (Mann Whitney test p = 0.69) (Additional file 1: Table 6). No significant treatment effect was found after comparing the STD at peak exercise between the second ETT on placebo with the ETT on liraglutide 1.8 mg irrespective of trial period (Wilcoxon matched-pairs signed rank test p = 0.571) (Fig. 3d).

### Secondary inducible ischaemia endpoints

#### Change in time to 0.1 mV STD

**Fig. 4:**
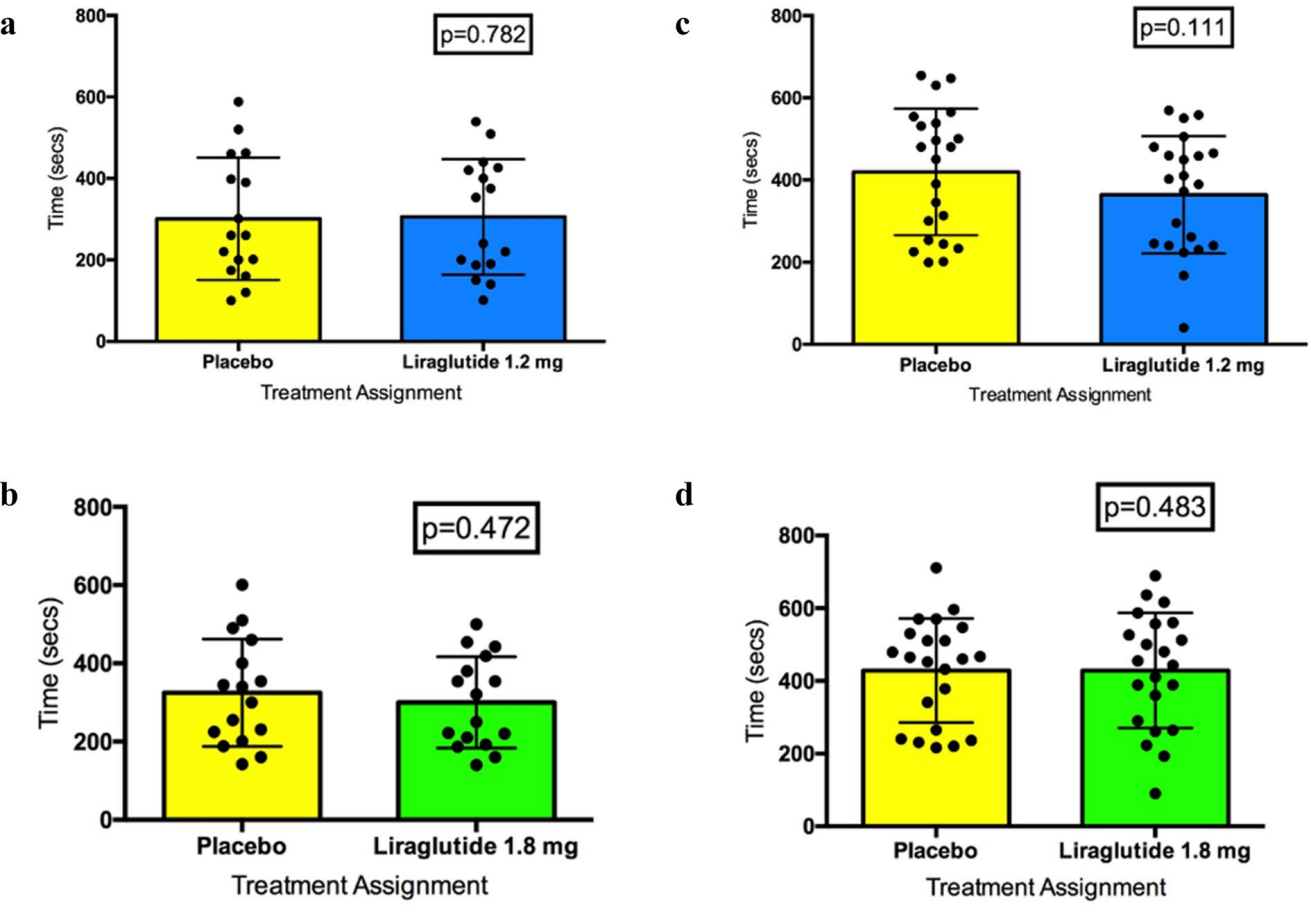
Secondary inducible ischaemia endpoints. **a** Time to 0.1 mV ST-segment depression ETT-1 placebo vs. ETT-3 1.2 mg liraglutide. **b** Time to 0.1 mV ST-segment depression ETT-2 placebo vs. ETT-4 1.8 mg liraglutide. **c** Time to maximum ST-segment depression ETT-1 placebo vs. ETT-3 1.2 mg liraglutide. **d** Time to maximum ST-segment depression ETT-2 placebo vs. ETT-4 1.8 mg liraglutide

Of the 22 patients who completed the protocol, 16 patients achieved STD ≥ 0.1 mV during serial exercise stress testing and were subsequently analysed. There was no carryover effect from ETT-1 to ETT-3 (Mann Whitney test p = 0.28). No significant treatment effect was observed between ETT-1 on placebo and ETT-3 on liraglutide 1.2 mg irrespective of trial period (Wilcoxon matched-pairs signed rank test p = 0.782) (Fig. 4a) (Additional file 1: Table S7).

There was no carryover effect from ETT-2 to ETT-4 (Mann Whitney test p = 0.279). No significant treatment effect was seen between ETT-2 on placebo ETT-4 on liraglutide 1.8 mg irrespective of trial period (Wilcoxon matched-pairs signed rank test p = 0.472) (Fig. 4b) (Additional file 1: Table S8).

#### Change in time to maximum STD

There was no carryover effect from ETT-1 to ETT-3 (Mann Whitney test p = 0.261). A paired comparison of the time to maximum STD between ETT-1 on placebo with ETT-3 on liraglutide 1.2 mg irrespective of trial period demonstrated no significant treatment effect (Wilcoxon matched-pairs signed rank test p = 0.111) (Fig. 4c) (Additional file 1: Table S9).

There was no carryover effect from ETT2 to ETT4 (Mann Whitney test p = 0.381). A paired comparison of the time to maximum STD between ETT-2 on placebo ETT-4 on liraglutide 1.8 mg irrespective of trial period, showed no significant treatment effect (Wilcoxon matched-pairs signed rank test p = 0.4826) (Fig. 4d) (Additional file 1: Table S10).

### Secondary exercise capacity endpoints

#### Change in total exercise time

**Fig. 5:**
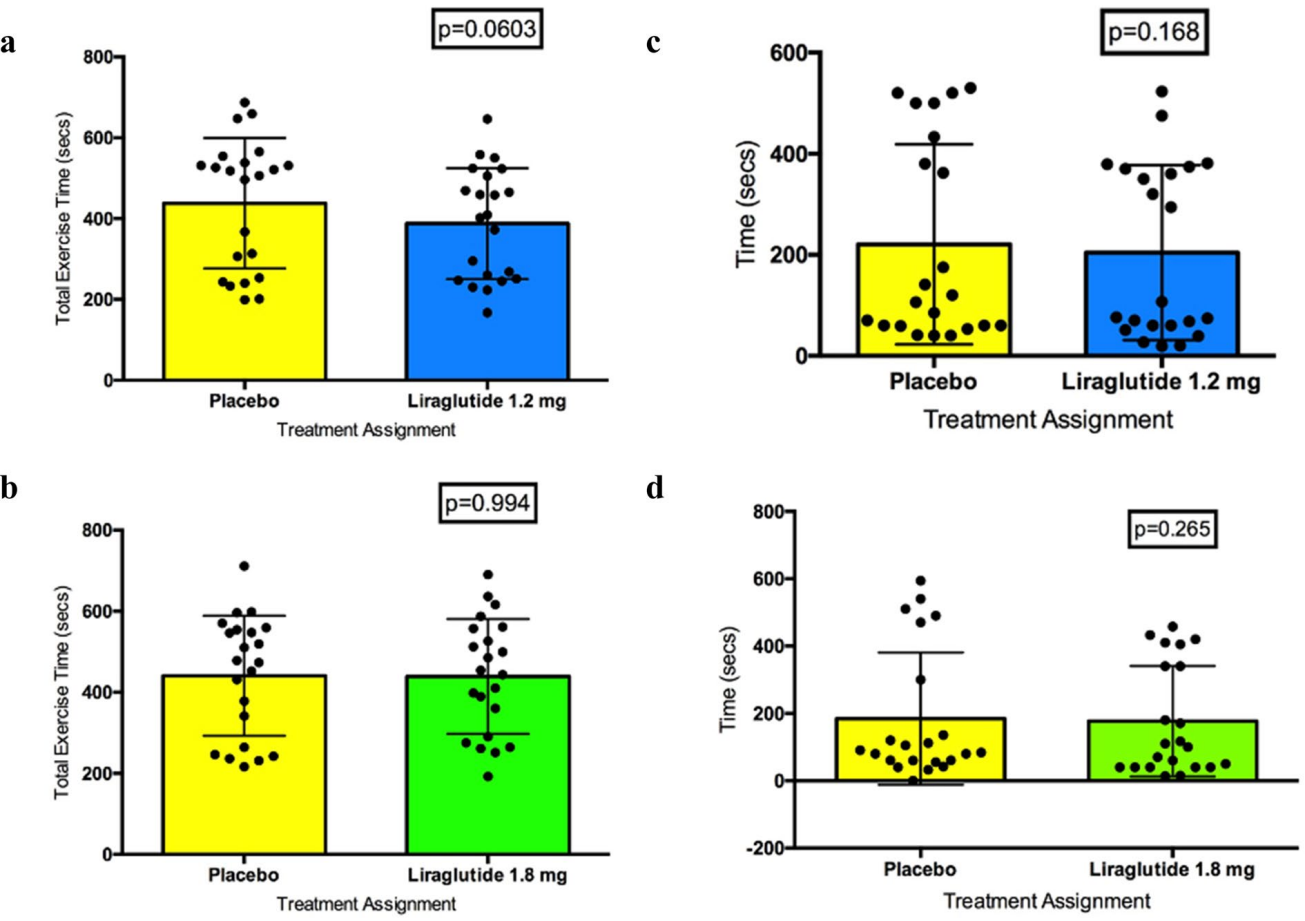
Secondary exercise capacity endpoints. **a** Total exercise time ETT-1 placebo vs. ETT-3 1.2 mg liraglutide. **b** Total exercise time ETT-2 placebo vs. ETT-4 1.8 mg liraglutide. **c** Recovery time to 0.05 mV ST-segment depression ETT-1 placebo vs. ETT-3 1.2 mg liraglutide. **d** Recovery time to 0.05 mV ST-segment depression ETT-2 placebo vs. ETT-4 1.8 mg liraglutide

There was no carryover effect from ETT-1 to ETT-3 (Mann Whitney test p = 0.72). No significant treatment effect was found between ETT-1 on placebo and ETT-3 on liraglutide 1.2 mg irrespective of trial period (Wilcoxon matched-pairs signed rank test p = 0.06) (Fig. 5a) (Additional file 1: Table S11).

There was no carryover effect from ETT-2 to ETT-4 (Mann Whitney test p = 0.46). No significant treatment effect was found between ETT-2 on placebo and ETT-4 on liraglutide 1.8 mg irrespective of trial period (Wilcoxon matched-pairs signed rank test p = 0.99) (Fig. 5b) (Additional file 1: Table S12).

#### Change in recovery time to 0.05 mV STD

There was no carryover effect from ETT-1 to ETT-3 (Mann Whitney test p = 0.54). A paired comparison of the recovery time to 0.05 mV STD between ETT-1 on placebo with ETT-3 on liraglutide 1.2 mg, irrespective of trial period, confirmed no significant treatment effect (Wilcoxon matched-pairs signed rank test p = 0.17) (Fig. 5c) (Additional file 1: Table S13).

There was no carryover effect from ETT-2 to ETT-4 (Mann Whitney test p = 0.314). A paired comparison of the recovery time to 0.05 mV STD directly between ETT-2 on placebo with ETT-4 on Liraglutide 1.8 mg, irrespective of trial period, also confirmed no significant treatment effect (Wilcoxon matched-pairs signed rank test p = 0.265) (Fig. 5d) (Additional file 1: Table S14).

### Secondary safety endpoints

#### Hypoglycaemia

Recruits were asked to take twice daily home blood glucose measurements (HBGM) throughout the course of the trial. From a possible 1848 measurements (i.e. 44 per day multiplied by 42 days), 1818 recordings were available for analysis: a response rate of 98.4%. There were no symptomatic episodes of hypoglycaemia recorded for any participant throughout the course of the trial [26]. Overall average blood glucose levels were significantly lower in the morning (Wilcoxon matched-pairs signed rank test p < 0.0001) and the afternoon (paired t-test p < 0.0001) during the liraglutide treatment phase versus placebo (Additional file 1: Fig. S1).

Random plasma glucose (RPG) was also checked at baseline and at each study visit. A repeated measures one-way ANOVA demonstrated a significant difference (p = 0.001) between mean RPG at baseline (5.8 ± 1.5 mmol/L) versus mean RPG after liraglutide (5.2 ± 0.7 mmol/L) versus mean RPG after placebo (5.7 ± 0.9 mmol/L). There was a significant difference when mean RPG after liraglutide was compared with either baseline or placebo separately. There was no significant difference between mean RPG at baseline versus mean RPG after placebo (Additional file 1: Fig. S2).

#### Renal function

A repeated measures one-way ANOVA demonstrated no significant difference in the mean serum creatinine at baseline (91.6 ± 24.6 μmol/L) versus after the placebo trial period (89.6 ± 24.4 μmol/L) versus after the liraglutide trial period (88.8 ± 21.0 μmol/L) (p = 0.46). Likewise, there was no significant difference in the estimated glomerular filtration rate at baseline (76.2 ± 21.1 mL/min/1.73 m^2^) versus after placebo (77.7 ± 18.8 mL/min/1.73 m^2^) versus after liraglutide (77.7 ± 18.6 mL/min/1.73 m^2^) (p = 0.451) (Additional file 1: Fig. S3).

#### Acute pancreatitis

There were no documented cases of acute pancreatitis during the trial. A repeated measures one-way ANOVA of serum amylase at baseline (68.1 ± 27.4 IU) versus mean amylase during placebo (75.1 ± 34.4 IU) versus mean amylase during liraglutide (77.6 ± 41.5 IU) showed no significant difference between the treatment periods (p = 0.06) (Additional file 1: Fig. S4).

### Assessment of chronic GLP-1 receptor activation

GLP-1Ra’s in general, and liraglutide in particular, have been shown to mediate indirect benefits on cardiovascular risk such as weight loss, improvements in lipid profile, and net reductions in blood pressure (BP) [27–35]. These parameters were readily available for measurement during the course of the 6-week protocol and were designed to act as surrogate markers of patient compliance with the blinded study drugs.

#### Weight

A repeated measures one-way ANOVA comparing weight at baseline (88.75 ± 16.5 kg) versus mean weight after placebo (88.11 ± 16.27 kg) versus mean weight after liraglutide (87.78 ± 16.86 kg) confirmed a significant difference (p = 0.0008) between mean weight at baseline versus mean weight after liraglutide. There was no difference between mean weight at baseline versus mean weight after placebo or mean weight after placebo versus mean weight after liraglutide (Additional file 1: Fig. S5).

#### Blood pressure

A repeated measures one-way ANOVA of mean systolic BP at baseline (134.4 ± 18.3 mmHg) versus mean systolic BP after placebo (139.2 ± 17.7 mmHg) versus mean systolic BP after liraglutide (137.1 ± 16.5 mmHg) showed no significant differences (p = 0.23) (Additional file 1: Fig. S6A).

A repeated measures one-way ANOVA of mean diastolic BP at baseline (77.8 ± 9.8 mmHg) versus mean diastolic BP after placebo (80.1 ± 12.8 mmHg) versus mean diastolic BP after liraglutide (81.6 ± 13.6 mmHg) also revealed no significant differences (p = 0.80) (Additional file 1: Fig. S6B).

#### Lipid profile

**Fig. 6:**
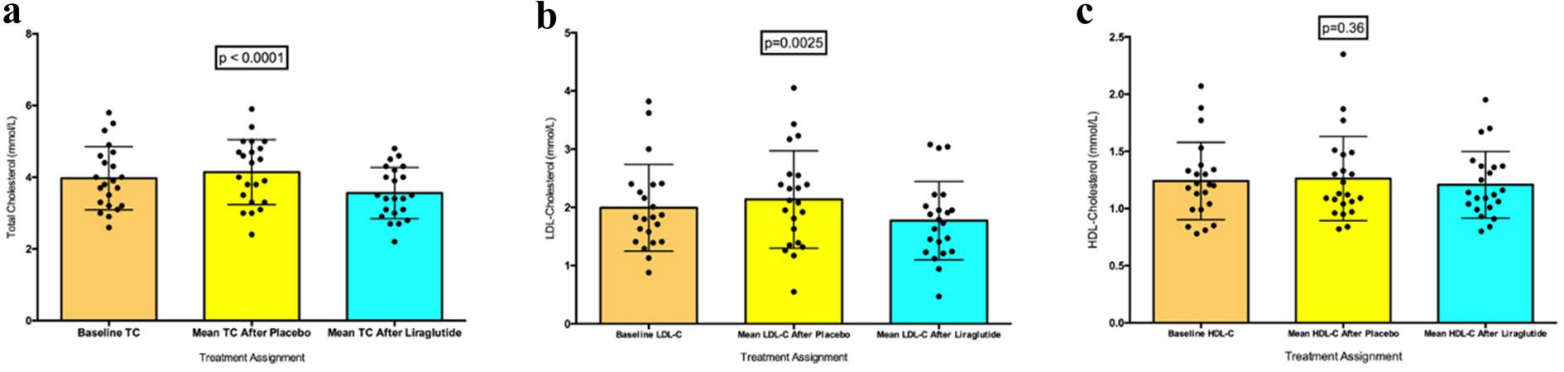
Changes in lipid profile constituents. **a** Mean total cholesterol. **b** Mean low density lipoprotein cholesterol (LDL-C). **c** Mean high density lipoprotein cholesterol (HDL-C)

Repeated measures one-way ANOVA of total cholesterol (TC) measurements throughout the trial demonstrated a significant difference (p < 0.0001). There was a significant difference seen between baseline TC (3.97 ± 0.88 mmol/L) versus TC after liraglutide (3.56 ± 0.71 mmol/L) and a significant difference between TC after placebo (4.14 ± 0.91 mmol/L) versus TC after liraglutide (Fig. 6a).

Repeated measures one-way ANOVA of low-density lipoprotein cholesterol (LDL-C) levels throughout the trial also revealed a significant difference between LDL-C after saline placebo (2.14 ± 0.84 mmol/L) versus LDL-C after liraglutide (1.77 ± 0.67 mmol/L) only (p = 0.003) (Fig. 6b).

Repeated measures one-way ANOVA of HDL-C measurements throughout the trial demonstrated no significant differences (p = 0.36) (Fig. 6c).

### Assessment of symptoms

#### Angina

Trial participants were given a patient diary and asked to note down any episodes of angina occurring throughout the study duration. Diary entries were recorded at every trial visit. The frequency of angina episodes were, however, relatively low (Additional file 1: Table S15). There was also no significant difference in frequency of angina on placebo when compared with liraglutide (Wilcoxon matched-pairs signed rank test p = 0.14) (Additional file 1: Fig. S7A).

#### Gastrointestinal adverse effects

Gastrointestinal (GI) upset is fairly common when commencing GLP-1Ra therapy. As with angina frequency, we asked all trial recruits to note down any episodes of nausea, vomiting, abdominal discomfort or diarrhoea in their diaries. Overall there was a relatively low incidence of GI symptoms (Additional file 1: Table S16). As expected, however, there was a significantly higher incidence of GI symptoms after liraglutide therapy compared with placebo (Wilcoxon matched-pairs signed rank test p = 0.008) (Additional file 1: Fig. S7B). These GI symptoms did not lead to the premature withdrawal of any patient from the trial.

## Discussion

To the best of our knowledge, we have shown for the first time in the literature that chronic GLP-1R activation, mediated by liraglutide, does not act in an anti-anginal capacity in a cohort of SIHD patients with obstructive CAD. Liraglutide did not enhance any parameters of haemodynamic performance measured during serial exercise testing. Liraglutide did not significantly augment indices of cardiac function such as the rate pressure product and did not attenuate the magnitude of ST-segment depression seen at peak exercise. There was no dose–response effect seen, such that neither the standard dose of 1.2 mg, or a higher dose of 1.8 mg, were able to provoke a significant anti-anginal or anti-ischaemic effect compared to saline placebo. Furthermore, liraglutide did not significantly reduce the number of angina episodes during daily activity when compared with placebo, although the overall incidence of angina in the cohort was relatively low. The validity of these findings are further reinforced by the relative parity noted in the percentage of THR achieved across all four of the serial ETTs. This would suggest there was no training effect acting as a potential confounder during the 6-week trial protocol. Recent evidence has emerged linking liraglutide to increased heart rate, reduced heart rate variability and a detrimental effect on sympathovagal balance [36–38]. In the present trial this putative haemodynamic effect was not observed.

We used a crossover design for the LIONESS trial so each patient served as their own control. This helps to avoid problems associated with the comparability of study and control groups and confounding variables such as age and gender since intra-individual rather than inter-individual differences were studied (Table 1). A comparison of treatments on the same subject is also expected to be more precise which means a smaller sample size could be utilised to achieve adequate statistical power. We were able to achieve our target sample size.

Moreover, crossover can be advantageous with respect to the power of the statistical test carried out to confirm the existence of a treatment effect. To exploit these advantages it is imperative to factor in a washout phase that is sufficiently long enough to rule out a carryover effect. The 2-week interval between ETTs from the first treatment period to the next is well over the accepted mark of ≥ 5 half-lives of the active agent under investigation. Indeed a carryover effect was not detected for any of the haemodynamic primary and secondary endpoints measured in the trial, which confirmed a suitable washout period had been incorporated.

We did, however, demonstrate the safety of using liraglutide in a predominantly non-diabetes population, reflected by the absence of symptomatic hypoglycaemia throughout the study. GLP-1Ra are known to mediate beneficial effects on lipid profile and stimulate weight loss [12, 39–41]. This positive modulation of cardiovascular risk was replicated in the trial with significant reductions in weight, total cholesterol and LDL-cholesterol recorded after the liraglutide phase of treatment. Modification of the lipid profile could be due to biological variation or a play of chance as opposed to a direct (or indirect) GLP-1-mediated effect. However, 21 out of 22 patients continued taking statins throughout the 6-week trial (Additional file 1: Table S1). This may reinforce the plausibility of the significant changes seen in the lipid profile.

We did not see any significant effects on BP. Again the short duration of active agent uptake may have been a factor here along with the temporary cessation of beta-blocker therapy in 20 participants across the 6 weeks [40, 42]. This may have negated any net GLP-1-mediated modulation of BP. Ultimately, we should be clear that the trial was not powered (nor designed) to detect differences in weight, lipid profile, or BP.

The GLP-1Ra’s have been studied in several large cardiovascular outcomes trials in patients with type 2 diabetes and elevated cardiovascular risk to demonstrate their safety. Trials of liraglutide (LEADER), exenatide (EXSCEL), lixisenatide (ELIXA), semaglutide (SUSTAIN-6), and albiglutide (HARMONY OUTCOMES) have all confirmed cardiovascular safety but interestingly, liraglutide, semaglutide and albiglutide have also significantly reduced the number of cardiovascular events [9, 10, 12–14]. Furthermore, in a post hoc analysis of the LEADER trial, liraglutide was shown to consistently reduce major cardiovascular events in patients with established multi-vessel and single vessel CAD [43]. A recent systematic review and meta-analysis of cardiovascular outcome trials, also confirmed the observation that treatment with GLP-1Ra’s in T2DM patients improves cardiovascular, mortality and kidney outcomes [44]. The mechanisms by which these beneficial cardiovascular effects are mediated remain elusive but are thought to be driven by their non-glycaemic effects [40]. The LIONESS trial would suggest the modulation of cardiovascular risk secondary to chronic GLP-1R activation is unlikely to be via an anti-anginal or anti-ischaemic effect, particularly in the context of SIHD. In acute coronary syndromes, however, small studies have shown liraglutide can improve myocardial salvage and ameliorate infarct size after ST-elevation myocardial infarction (STEMI) and left ventricular function following STEMI and non-STEMI [45–47]. Similar effects have been noted for exenatide in STEMI [17, 18].

### Limitations

The duration of liraglutide therapy may be a limiting factor. A more sustained period of active agent may have stimulated greater improvements in exercise haemodynamics. Adequate compliance with study agents was strongly encouraged and the number of empty syringes were recorded at every trial visit. Significant differences in HBGM, weight, lipid profile and gastrointestinal side effects would suggest participants were compliant with their study agents and biologically meaningful GLP-1R activation was achieved. Moreover extending the trial beyond 6 weeks would have been logistically difficult, may have allowed a training effect to emerge, and could have resulted in more recruits withdrawing from the trial prematurely to proceed to elective coronary revascularisation.

The diagnostic value of exercise tolerance testing as a modality to detect reversible myocardial ischaemia in the context of current guideline-mandated algorithms, might also be questioned. We accept exercise stress testing has relatively poor sensitivity (mean 67%) and specificity (mean 72%) depending on which published report you read. In the context of the LIONESS trial, however, this was acceptable given that all patients were required to have established CAD confirmed by coronary angiography. The determination of the functional significance of every coronary stenosis deemed obstructive angiographically was not a prerequisite for trial inclusion, but this potential flaw was countered by the need for evidence of inducible ischaemia from a baseline ETT for every trial participant.

Pharmacological agents used to replicate exercise stress are more likely to achieve ≥ 85% of THR during stress echocardiography, stress perfusion cardiac magnetic resonance imaging and nuclear myocardial perfusion scanning. These imaging modalities study left ventricular function and regional wall motion (so can therefore better localise the distribution of ischaemia) but not the exercise haemodynamics via specific electrophysiological parameters we were investigating. Moreover exercise is preferred over pharmacological testing because exercise can induce higher physiological stress and better correlation between symptoms and physical work capacity than that achieved by pharmacological testing. Moreover, recruits achieved on average > 81% of their THR during the trial which validates the ability of serial exercise testing to induce reproducible myocardial ischaemia.

## Conclusions

In the LIONESS trial, chronic GLP-1R activation mediated by liraglutide, did not improve exercise haemodynamics or augment established indices of cardiac function in an all comer population of SIHD patients with known obstructive CAD and inducible myocardial ischaemia. Liraglutide did not cause symptomatic hypoglycaemia (in a predominantly non-diabetes trial cohort) and significantly reduced weight and stimulated net improvements in lipid profile, in line with the known effects of injectable GLP-1R agonists.

## Supplementary Information

**Additional file 1: LIONESS Trial Supplementary Material**

## Data Availability

All data generated or analysed during this study are included in this published article and associated additional files.

## Abbreviations

T2DM: Type 2 diabetes mellitus
DPP-4: Dipeptidyl peptidase-4
GLP-1Ra: Glucagon-like peptide-1 receptor agonist
MI: Myocardial infarction
ETT: Exercise treadmill test
CAD: Coronary artery disease
PCI: Percutaneous coronary intervention
CABG: Coronary artery bypass graft
ECG: Electrocardiogram
BP: Blood pressure
HBGM: Home blood glucose measurements
STD: ST-segment depression
RPP: Rate pressure product
