# Supplementary material for: Liraglutide to Improve corONary haemodynamics during Exercise streSS (LIONESS): a double-blind randomised placebo-controlled crossover trial
Source: Diabetol Metab Syndr. 2021 Feb 12;13:17. doi: 10.1186/s13098-021-00635-6 (PMC7881597; doi:10.1186/s13098-021-00635-6)

**Online Supplemental Material**

**LIONESS Trial Inclusion Criteria**

- Men and women aged 18-80
- Patients must be able to walk confidently on an exercise treadmill
- Patients must have a recent/baseline abnormal exercise tolerance test demonstrating >0.1 mV of planar or down-sloping ST-segment depression
- Patients must have angiographic evidence of a >70% stenosis in a main epicardial coronary artery, with or without coronary stenoses elsewhere
- Patients must have a normal resting ECG in sinus rhythm without bundle branch aberration or other conduction disturbance
- Patients must have preserved left ventricular systolic function (ejection fraction ≥40%)

**LIONESS Trial Exclusion Criteria**

- An abnormal resting ECG including atrial fibrillation, bundle brunch aberration or other conduction disturbance
- Pre-existing significant left ventricular systolic dysfunction (ejection fraction <40%)
- Pre-existing ischaemic or non-ischaemic cardiomyopathy
- Pre-existing haemodynamically significant valvular heart disease
- Inability to safely negotiate an exercise treadmill independently
- Patients with Type 1 diabetes mellitus
- Patients with Type 2 diabetes mellitus taking oral and/or subcutaneous anti-diabetic therapy
- Patients with a personal or family history of medullary thyroid carcinoma
- Patients with Multiple Endocrine Neoplasia syndrome type 2
- Patients with acute renal failure or deteriorating renal function

**Guidelines employed to analyse exercise stress tests**

The following guidelines were employed to ensure uniformity of serial exercise stress test analysis following completion of the LIONESS Trial:

- Measure ST-segment depression relative to original baseline at start of the exercise test – not necessarily the isoelectric line.
- Any ST-segment deviation in a positive direction should be excluded from the analysis.
- The first time point at which recovery to 0.05 mV ST-segment depression is achieved should be taken as the time to recovery; irrespective of whether there is a biphasic pattern of ST-segment deviation occurring in the recovery phase.
- If the ST-segment is already below the isoelectric line measure recovery to 0.05 mV relative to the original baseline.
- If the ST-segment is **below** the isoelectric line at baseline and a return to 0.05 mV relative to that baseline is not achieved then use the time taken to get back to that original baseline.
- If the ST-segment is **above** the isoelectric line at baseline take recovery as 0.05 mV relative to the original baseline – again not necessarily relative to the isoelectric line.
- For those patients ultimately achieving their maximum ST-segment depression in recovery – use the peak exercise time as the time to max ST-segment depression.
- For those patients not achieving 0.1 mV ST-segment depression - use 0.05 mV ST-segment depression as a cut-off.

All exercise tests were analysed by two investigators independently of each other and results compared thereafter. All exercise tests were analysed and the results documented before the trial was un-blinded.

| ***Group A** | **Cardiovascular medications continued** | **Cardiovascular medications temporarily withheld** |
| --- | --- | --- |
| LIONESS01 | Aspirin 75 mg od, Ramipril 5 mg od, Amlodipine 5 mg od, Atorvastatin 40 mg od | Bisoprolol 5 mg od  Atorvastatin 40 mg od |
| LIONESS02 | Aspirin 75 mg od, Losartan 25 mg od, Simvastatin 40 mg od, GTN spray 2 puffs PRN | Atenolol 50 mg  Isotard XL 30 mg od |
| LIONESS04 | Aspirin 75 mg od, Losartan 100 mg od, Indapamide 2.5 mg od, Atorvastatin 20 mg od, GTN spray 2 puffs PRN | Bisoprolol 2.5 mg od  Diltiazem 240 mg od |
| LIONESS06 | Aspirin 75 mg od, Clopidogrel 75 mg od, Enalapril 25 mg od, Amlodipine 5 mg od, Atorvastatin 40 mg od | Atenolol 50 mg od  Isosorbide mononitrate MR 120 mg od |
| LIONESS09 | Clopidogrel 75 mg od, Irbesartan/Hydrochlorothiazide 300/12.5 1 tablet od, Atorvastatin 10 mg od, GTN spray 2 puffs PRN | Bisoprolol 2.5 mg od |
| LIONESS10 | Aspirin 75 mg od, Irbesartan 300 mg od, Lercanidipine 10 mg od, Atorvastatin 10 mg od | Bisoprolol 5 mg od |
| LIONESS12 | Aspirin 75 mg od, Ramipril 2.5 mg od, Atorvastatin 80 mg nocte, GTN spray 2 puff PRN | Bisoprolol 1.25 mg od |
| LIONESS13 | Aspirin 75 mg od, Amlodipine 5 mg od, Simvastatin 20 mg od, GTN spray 2 puffs PRN | Nebivolol 5 mg od  Isosorbide mononitrate 40 mg (am) 20 mg (pm) |
| LIONESS17 | Aspirin 75 mg od, Atorvastatin 20 mg od, GTN spray 2 puffs PRN | Monomil XL 30 mg od |
| LIONESS20 | Aspirin 75 mg od, Clopidogrel 75 mg od, Losartan 25 mg od, Amlodipine 10 mg od, Pravastatin 20 mg od | Bisoprolol 1.25 mg od |
| LIONESS23 | Aspirin 75 mg od, Simvastatin 40 mg od, GTN spray 2 puffs PRN | Nicorandil 10 mg bd |
| LIONESS24 | Aspirin 75 mg od, Ticagrelor 90 mg bd, Ramipril 2.5 mg od, Atorvastatin 10 mg od, GTN spray 2 puffs PRN | Bisoprolol 2.5 mg od |
| ***Group B** | **Cardiovascular medications continued** | **Cardiovascular medications temporarily withheld** |
| LIONESS03 | Aspirin 75 mg od, Prasugrel 10 mg od, Ramipril 2.5 mg od, Atorvastatin 40 mg od, GTN spray 2 puffs PRN | Bisoprolol 2.5 mg od |
| LIONESS05 | Aspirin 75 mg od, Ramipril 2.5 mg od, Atorvastatin 40 mg od, GTN spray 2 puffs PRN | Bisoprolol 5 mg od  Isosorbide mononitrate 10 mg bd |
| LIONESS07 | Aspirin 75 mg od, Clopidogrel 75 mg od, Ramipril 10 mg od, Indapamide 1.5 mg od, Atorvastatin 40 mg od | Bisoprolol 10 mg od  Isotard XL 25 mg od |
| LIONESS08 | Aspirin 75 mg od, Clopidogrel 75 mg od, Perindopril 2 mg od, Amlodipine 5 mg od, Simvastatin 40 mg nocte | Bisoprolol 5 mg od  Isotard XL 60 mg od |
| LIONESS11 | Aspirin 75 mg od, Simvastatin 20 mg od | Bisoprolol 2.5 mg od  Isotard XL 25 mg od |
| LIONESS14 | Aspirin 75 mg od, Irbasartan 150 mg od, Amlodipine 5 mg od, Bendroflumethiazide 2.5 mg od, Atorvastatin 80 mg od, GTN spray 2 puffs PRN | Bisoprolol 1.25 mg od  Nicorandil 20 mg bd |
| LIONESS15 | Aspirin 75 mg od, Clopidogrel 75 mg od, Ramipril 2.5 mg od, Atorvastatin 40 mg od, GTN spray 2 puffs PRN | Bisoprolol 2.5 mg od |
| LIONESS16 | Aspirin 75 mg od, Clopidogrel 75 mg od, Enalapril 10 mg od, Bendroflumethiazide 2.5 mg od, Atorvastatin 80 mg od, GTN spray 2 puffs PRN | Bisoprolol 2.5 mg od |
| LIONESS18 | Aspirin 75 mg od, Clopidogrel 75 mg od, Ramipril 1.25 mg od, Atorvastatin 80 mg od, GTN spray 2 puffs PRN | Bisoprolol 5 mg od |
| LIONESS19 | Aspirin 75 mg od, Amlodipine 10 mg od | None |
| *Group A=saline then liraglutide and Group B=liraglutide then saline treatment sequences after un-blinding of treatment allocation  Key: bd=twice daily; GTN=glyceryl trinitrate; mg=milligram; MR=modified release; od=once daily; PRN=pro re nata (as required); XL=extended release | | |

**Supplemental Table 1 LIONESS trial patient cardiovascular medications**

**Supplemental Table 2**

**Percentage of target heart rate achieved by trial participants during serial exercise tolerance testing**

| ***Group A** | **Baseline ETT** | **ETT on placebo** | **ETT on placebo** | **ETT on**  **Liraglutide 1.2 mg** | **ETT on**  **Liraglutide 1.8 mg** |
| --- | --- | --- | --- | --- | --- |
| LIONESS01 | 59 | 102 | 94 | 87 | 89 |
| LIONESS02 | 89 | 88 | 87 | 91 | 92 |
| LIONESS04 | 78 | 92 | 94 | 92 | 96 |
| LIONESS06 | 69 | 77 | 70 | 72 | 76 |
| LIONESS09 | 74 | 76 | 75 | 75 | 68 |
| LIONESS10 | 70 | 83 | 86 | 86 | 80 |
| LIONESS12 | 86 | 87 | 82 | 78 | 78 |
| LIONESS13 | 56 | 67 | 66 | 75 | 70 |
| LIONESS17 | 80 | 75 | 86 | 74 | 80 |
| LIONESS20 | 95 | 97 | 90 | 99 | 93 |
| LIONESS23 | 78 | 83 | 84 | 85 | 89 |
| LIONESS24 | 86 | 84 | 81 | 76 | 76 |
| ***Group B** | **Baseline ETT** | **ETT on placebo** | **ETT on placebo** | **ETT on**  **Liraglutide 1.2 mg** | **ETT on**  **Liraglutide 1.8 mg** |
| LIONESS03 | 83 | 96 | 92 | 98 | 101 |
| LIONESS05 | 62 | 63 | 64 | 69 | 68 |
| LIONESS07 | 101 | 92 | 95 | 93 | 92 |
| LIONESS08 | 74 | 76 | 79 | 74 | 83 |
| LIONESS11 | 62 | 79 | 73 | 71 | 77 |
| LIONESS14 | 63 | 80 | 78 | 74 | 76 |
| LIONESS15 | 62 | 70 | 66 | 87 | 71 |
| LIONESS16 | 74 | 64 | 66 | 71 | 60 |
| LIONESS18 | 70 | 77 | 81 | 78 | 75 |
| LIONESS19 | 78 | 78 | 80 | 80 | 80 |
| **Mean** | 74.95 | 81.18 | 80.41 | 81.14 | 80.45 |
| **Standard deviation** | ±11.93 | ±10.53 | ±9.82 | ±9.20 | ±10.41 |
| **Lower 95%**  **confidence interval** | 69.67 | 76.51 | 76.06 | 77.06 | 75.84 |
| **Upper 95%**  **confidence interval** | 80.24 | 85.85 | 84.76 | 85.22 | 85.07 |
| *Results shown indepedent of treatment period or sequence. | | | | | |

**Supplemental Table 3**

**Rate pressure product at 0.1 mV ST-segment depression for ETT-1 versus ETT-3**

A Mann Whitney test for a non-parametric distribution confirmed no significant difference (p= 0.1802) between the sum totals of Groups A and B (column ETT1 + ETT3), thereby excluding a significant carryover effect.

| **GROUP A PATIENT** | **ETT1 Placebo RPP** | **ETT3 Lira1.2 RPP** | **ETT1 + ETT3** | **ETT1 - ETT3** |
| --- | --- | --- | --- | --- |
| LIONESS01 | 32856 | 19600 | 52456 | 13256 |
| LIONESS02 | 22572 | 21809 | 44381 | 763 |
| LIONESS04 | 34100 | 29376 | 63476 | 4724 |
| LIONESS06 | 16872* | 15301* | 32173 | 1571 |
| LIONESS09 | 22000* | 20608* | 42608 | 1392 |
| LIONESS10 | 22698 | 23353 | 46051 | -655 |
| LIONESS12 | 23975 | 18327 | 42302 | 5648 |
| LIONESS13 | 17472* | 17066* | 34538 | 406 |
| LIONESS17 | 23728 | 20909 | 44637 | 2819 |
| LIONESS20 | 19404 | 17956 | 37360 | 1448 |
| LIONESS23 | 20900 | 18139 | 39039 | 2761 |
| LIONESS24 | 24120* | 19920* | 44040 | 4200 |
|  |  | **Mean =** | **43588.42** | **3194.42** |
|  |  | **SD =** | **8292.79** | **3672.68** |
| **GROUP B PATIENT** | **ETT1 Lira1.2 RPP** | **ETT3 Placebo RPP** | **ETT1 + ETT3** | **ETT1 - ETT3** |
| LIONESS03 | 20286 | 25192 | 45478 | -4906 |
| LIONESS05 | 20453 | 19186* | 39639 | 1267 |
| LIONESS07 | 30000* | 26344* | 56344 | 3656 |
| LIONESS08 | 22260* | 21942* | 44202 | 318 |
| LIONESS11 | 16650* | 18300* | 34950 | -1650 |
| LIONESS14 | 14868 | 13209 | 28077 | 1659 |
| LIONESS15 | 16688 | 16008 | 32696 | 680 |
| LIONESS16 | 18297 | 16638 | 34935 | 1659 |
| LIONESS18 | 19320 | 17854 | 37174 | 1466 |
| LIONESS19 | 17545 | 16402 | 33947 | 1143 |
|  |  | **Mean =** | **38744.20** | **529.20** |
|  |  | **SD =** | **8099.66** | **2321.07** |
| *RPP at peak exercise when 0.1 mV ST-segment depression was not achieved  Key: ETT=exercise treadmill test; RPP=rate pressure product; Lira=liraglutide; SD=standard deviation | | | | |

**Supplemental Table 4**

**Rate pressure product at 0.1 mV ST-segment depression for ETT-2 versus ETT-4**

There was no significant carryover effect from ETT2 to ETT4 for either group of trial participants as evidenced by a Mann Whitney test (p=0.381) of the sum of RPP values (column ETT2 + ETT4) from both treatment sequences.

| **GROUP A PATIENT** | **ETT2 Placebo RPP** | **ETT4 Lira1.8 RPP** | **ETT2 + ETT4** | **ETT2 - ETT4** |
| --- | --- | --- | --- | --- |
| LIONESS01 | 24871 | 25929 | 50800 | -1058 |
| LIONESS02 | 22127 | 21774 | 43901 | 353 |
| LIONESS04 | 35802 | 25800 | 61602 | 10002 |
| LIONESS06 | 16478* | 16385* | 32863 | 93 |
| LIONESS09 | 20680 | 16320* | 37000 | 4360 |
| LIONESS10 | 23115 | 21692* | 44807 | 1423 |
| LIONESS12 | 25740 | 19264 | 45004 | 6476 |
| LIONESS13 | 16625* | 16728* | 33353 | -103 |
| LIONESS17 | 20844 | 12672 | 33516 | 8172 |
| LIONESS20 | 19312 | 17666 | 36978 | 1646 |
| LIONESS23 | 18437 | 20790 | 39227 | -2353 |
| LIONESS24 | 23875* | 21538* | 45413 | 2337 |
|  |  | **Average =** | **42038.67** | **2612.33** |
|  |  | **SD =** | **8431.38** | **3847.95** |
| **GROUP B PATIENT** | **ETT 2 Lira1.8 RPP** | **ETT 4 Placebo RPP** | **ETT2 + ETT4** | **ETT2 - ETT4** |
| LIONESS03 | 19856 | 17316 | 37172 | 2540 |
| LIONESS05 | 20010* | 18144 | 38154 | 1866 |
| LIONESS07 | 27448 | 18352 | 45800 | 9096 |
| LIONESS08 | 25155* | 22890* | 48045 | 2265 |
| LIONESS11 | 20880* | 19372* | 40252 | 1508 |
| LIONESS14 | 13668 | 13081 | 26749 | 587 |
| LIONESS15 | 17710 | 13312 | 31022 | 4398 |
| LIONESS16 | 15540 | 17100 | 32640 | -1560 |
| LIONESS18 | 17741 | 20740 | 38481 | -2999 |
| LIONESS19 | 16698 | 22264 | 38962 | -5566 |
|  |  | **Mean =** | **37727.70** | **1213.50** |
|  |  | **SD =** | **6428.63** | **4047.55** |
| *RPP at peak exercise when 0.1 mV ST-segment depression was not achieved  Key: ETT=exercise treadmill test; RPP=rate pressure product; Lira=liraglutide; SD=standard deviation | | | | |

**Supplemental Table 5**

**Change in degree of ST-segment depression at peak exercise for ETT-1 versus ETT-3**

There was no significant carryover effect from ETT1 to ETT3 comparing the sum of the values over both treatment periods between Groups A and B (Mann Whitney test p=0.759).

| **GROUP A PATIENT** | **ETT1 Placebo STD (mm)** | **ETT3 Lira1.2 STD (mm)** | **ETT1 + ETT3** | **ETT1 - ETT3** |
| --- | --- | --- | --- | --- |
| LIONESS01 | 1.30 | 0.00 | 1.30 | 1.30 |
| LIONESS02 | 1.95 | 1.75 | 3.70 | 0.20 |
| LIONESS04 | 1.65 | 1.20 | 2.85 | 0.45 |
| LIONESS06 | 0.80 | 0.75 | 1.55 | 0.05 |
| LIONESS09 | 0.00 | 0.00 | 0.00 | 0.00 |
| LIONESS10 | 0.40 | 1.10 | 1.50 | -0.70 |
| LIONESS12 | 3.30 | 4.55 | 7.85 | -1.25 |
| LIONESS13 | 0.50 | 0.45 | 0.95 | 0.05 |
| LIONESS17 | 1.30 | 1.80 | 3.10 | -0.50 |
| LIONESS20 | 1.50 | 0.70 | 2.20 | 0.80 |
| LIONESS23 | 2.90 | 2.80 | 5.70 | 0.10 |
| LIONESS24 | 0.40 | 0.45 | 0.85 | -0.05 |
|  |  | **Mean =** | **2.63** | **0.04** |
|  |  | **SD =** | **2.24** | **0.67** |
| **GROUP B PATIENT** | **ETT1 Lira1.2 STD (mm)** | **ETT3 Placebo STD (mm)** | **ETT1 + ETT3** | **ETT1 - ETT3** |
| LIONESS03 | 1.85 | 2.25 | 4.10 | -0.40 |
| LIONESS05 | 1.45 | 0.80 | 2.25 | 0.65 |
| LIONESS07 | 0.70 | 0.70 | 1.40 | 0.00 |
| LIONESS08 | 0.00 | 0.00 | 0.00 | 0.00 |
| LIONESS11 | 0.60 | 0.60 | 1.20 | 0.00 |
| LIONESS14 | 1.50 | 2.70 | 4.20 | -1.20 |
| LIONESS15 | 1.30 | 1.00 | 2.30 | 0.30 |
| LIONESS16 | 1.05 | 0.90 | 1.95 | 0.15 |
| LIONESS18 | 1.60 | 1.40 | 3.00 | 0.20 |
| LIONESS19 | 1.60 | 2.10 | 3.70 | -0.50 |
|  |  | **Mean =** | **2.41** | **-0.08** |
|  |  | **SD =** | **1.36** | **0.51** |
| Key: ETT=exercise treadmill test; Lira=liraglutide; mm=millimetres; SD=standard deviation; STD=ST-segment depression | | | | |

**Supplemental Table 6**

**Change in degree of ST-segment depression at peak exercise for ETT-2 versus ETT-4**

There was no significant carryover effect from ETT2 to ETT4 comparing the sum of the values over both treatment periods between Groups A and B (Mann Whitney test p=0.688).

| **GROUP A PATIENT** | **ETT2 Placebo STD (mm)** | **ETT4 Lira1.8 STD (mm)** | **ETT2 + ETT4** | **ETT2 - ETT4** |
| --- | --- | --- | --- | --- |
| LIONESS01 | 1.50 | 1.10 | 2.60 | 0.40 |
| LIONESS02 | 1.85 | 2.15 | 4.00 | -0.30 |
| LIONESS04 | 1.60 | 1.65 | 3.25 | -0.05 |
| LIONESS06 | 0.80 | 0.50 | 1.30 | 0.30 |
| LIONESS09 | 1.40 | 0.50 | 1.90 | 0.90 |
| LIONESS10 | 1.15 | 0.95 | 2.10 | 0.20 |
| LIONESS12 | 1.40 | 2.50 | 3.90 | -1.10 |
| LIONESS13 | 0.30 | 0.15 | 0.45 | 0.15 |
| LIONESS17 | 2.60 | 3.30 | 5.90 | -0.70 |
| LIONESS20 | 0.90 | 1.85 | 2.75 | -0.95 |
| LIONESS23 | 2.70 | 2.80 | 5.50 | -0.10 |
| LIONESS24 | 0.30 | 0.40 | 0.70 | -0.10 |
|  |  | **Mean =** | **2.86** | **-0.11** |
|  |  | **SD =** | **1.74** | **0.58** |
| **GROUP B PATIENT** | **ETT2 Lira1.8 STD (mm)** | **ETT4 Placebo STD (mm)** | **ETT2 + ETT4** | **ETT2 - ETT4** |
| LIONESS03 | 2.35 | 2.35 | 4.70 | 0.00 |
| LIONESS05 | 0.90 | 0.90 | 1.80 | 0.00 |
| LIONESS07 | 1.00 | 0.60 | 1.60 | 0.40 |
| LIONESS08 | 0.00 | 0.00 | 0.00 | 0.00 |
| LIONESS11 | 0.60 | 0.80 | 1.40 | -0.20 |
| LIONESS14 | 2.50 | 3.40 | 5.90 | -0.90 |
| LIONESS15 | 1.40 | 1.10 | 2.50 | 0.30 |
| LIONESS16 | 1.30 | 1.20 | 2.50 | 0.10 |
| LIONESS18 | 1.20 | 1.30 | 2.50 | -0.10 |
| LIONESS19 | 1.90 | 1.60 | 3.50 | 0.30 |
|  |  | **Mean =** | **2.64** | **-0.01** |
|  |  | **SD =** | **1.70** | **0.37** |
| Key: ETT=exercise treadmill test; Lira=liraglutide; mm=millimetres; SD=standard deviation; STD=ST-segment depression | | | | |

**Supplemental Table 7**

**Time to 0.1 mV ST-segment depression during ETT-1 versus ETT-3**

| **GROUP A PATIENT** | **ETT1 Placebo Time to 1mm STD (secs)** | **ETT3 Lira1.2 Time to 1mm STD (secs)** | **ETT1 + ETT3** | **ETT1 - ETT3** |
| --- | --- | --- | --- | --- |
| LIONESS01 | 301 | 426 | 727 | -125 |
| LIONESS02 | 174 | 187 | 361 | -13 |
| LIONESS04 | 220 | 200 | 420 | 20 |
| LIONESS10 | 460 | 440 | 900 | 20 |
| LIONESS12 | 120 | 101 | 221 | 19 |
| LIONESS17 | 160 | 220 | 380 | -60 |
| LIONESS20 | 260 | 509 | 769 | -249 |
| LIONESS23 | 200 | 190 | 390 | 10 |
|  |  | **Mean =** | **521** | **-47.25** |
|  |  | **SD =** | **242.14** | **96.21** |
| **GROUP B PATIENT** | **ETT1 Lira1.2 Time to 1mm STD (secs)** | **ETT3 Placebo Time to 1mm STD (secs)** | **ETT1 + ETT3** | **ETT1 - ETT3** |
| LIONESS03 | 353 | 462 | 815 | -109 |
| LIONESS05 | 150 | 201 | 351 | -51 |
| LIONESS07 | 539 | 588 | 1127 | -49 |
| LIONESS14 | 420 | 398 | 818 | 22 |
| LIONESS15 | 375 | 390 | 765 | -15 |
| LIONESS16 | 400 | 520 | 920 | -120 |
| LIONESS18 | 240 | 260 | 500 | -20 |
| LIONESS19 | 140 | 100 | 240 | 40 |
|  |  | **Mean =** | **692** | **-37.75** |
|  |  | **SD =** | **301.19** | **56.81** |
| Key: ETT=exercise treadmill test; Lira=liraglutide; secs=seconds; SD=standard deviation; STD=ST-segment depression | | | | |

**Supplemental Table 8**

**Time to 0.1 mV ST-segment depression during ETT-2 versus ETT-4**

| **GROUP A PATIENT** | **ETT 2 Placebo Time to 1mm STD (secs)** | **ETT 4 Lira1.8 Time to 1mm STD (secs)** | **ETT2 + ETT4** | **ETT2 - ETT4** |
| --- | --- | --- | --- | --- |
| LIONESS01 | 345 | 354 | 699 | -9 |
| LIONESS02 | 188 | 187 | 375 | 1 |
| LIONESS04 | 201 | 210 | 411 | -9 |
| LIONESS10 | 400 | 454 | 854 | -54 |
| LIONESS12 | 340 | 140 | 480 | 200 |
| LIONESS17 | 142 | 222 | 364 | -80 |
| LIONESS20 | 510 | 321 | 831 | 189 |
| LIONESS23 | 225 | 220 | 445 | 5 |
|  |  | **Mean =** | **557.38** | **30.38** |
|  |  | **SD =** | **204.80** | **105.45** |
| **GROUP B PATIENT** | **ETT 2 Lira1.8 Time to 1mm STD (secs)** | **ETT 4 Placebo Time to 1mm STD (secs)** | **ETT2 + ETT4** | **ETT2 - ETT4** |
| LIONESS03 | 443 | 460 | 903 | -17 |
| LIONESS05 | 192 | 231 | 423 | -39 |
| LIONESS07 | 500 | 601 | 1101 | -101 |
| LIONESS14 | 354 | 300 | 654 | 54 |
| LIONESS15 | 380 | 354 | 734 | 26 |
| LIONESS16 | 419 | 490 | 909 | -71 |
| LIONESS18 | 250 | 255 | 505 | -5 |
| LIONESS19 | 160 | 160 | 320 | 0 |
|  |  | **Mean =** | **693.63** | **-19.13** |
|  |  | **SD =** | **269.34** | **50.35** |
| Key: ETT=exercise treadmill test; Lira=liraglutide; secs=seconds; SD=standard deviation; STD=ST-segment depression | | | | |

**Supplemental Table 9**

**Time to maximum ST-segment depression during ETT-1 versus ETT-3**

| **GROUP A PATIENT** | **ETT1 Placebo Time to max STD (secs)** | **ETT3 Lira1.2 Time to max STD (secs)** | **ETT1 + ETT3** | **ETT1 - ETT3** |
| --- | --- | --- | --- | --- |
| LIONESS01 | 345 | 372 | 717 | -27 |
| LIONESS02 | 300 | 389 | 689 | -89 |
| LIONESS04 | 233 | 223 | 456 | 10 |
| LIONESS06 | 496 | 550 | 1046 | -54 |
| LIONESS09 | 480 | 40 | 520 | 440 |
| LIONESS10 | 480 | 449 | 929 | 31 |
| LIONESS12 | 244 | 295 | 539 | -51 |
| LIONESS13 | 554 | 558 | 1112 | -4 |
| LIONESS17 | 199 | 240 | 439 | -41 |
| LIONESS20 | 450 | 465 | 915 | -15 |
| LIONESS23 | 500 | 410 | 910 | 90 |
| LIONESS24 | 225 | 240 | 465 | -15 |
|  |  | **Mean =** | **728.08** | **22.92** |
|  |  | **SD =** | **245.76** | **139.08** |
| **GROUP B PATIENT** | **ETT1 Lira1.2 Time to max STD (secs)** | **ETT3 Placebo Time to max STD (secs)** | **ETT1 + ETT3** | **ETT1 - ETT3** |
| LIONESS03 | 569 | 630 | 1199 | -61 |
| LIONESS05 | 167 | 201 | 368 | -34 |
| LIONESS07 | 459 | 538 | 997 | -79 |
| LIONESS08 | 245 | 531 | 776 | -286 |
| LIONESS11 | 458 | 654 | 1112 | -196 |
| LIONESS14 | 480 | 647 | 1127 | -167 |
| LIONESS15 | 505 | 390 | 895 | 115 |
| LIONESS16 | 402 | 565 | 967 | -163 |
| LIONESS18 | 261 | 313 | 574 | -52 |
| LIONESS19 | 230 | 253 | 483 | -23 |
|  |  | **Mean =** | **849.80** | **-94.60** |
|  |  | **SD =** | **289.28** | **111.93** |
| Key: ETT=exercise treadmill test; Lira=liraglutide; secs=seconds; SD=standard deviation; STD=ST-segment depression | | | | |

**Supplemental Table 10**

**Time to maximum ST-segment depression during ETT-2 versus ETT-4**

| **GROUP A PATIENT** | **ETT2 Placebo Time to max STD (secs)** | **ETT4 Lira1.8 Time to max STD (secs)** | **ETT2 + ETT4** | **ETT2 - ETT4** |
| --- | --- | --- | --- | --- |
| LIONESS01 | 378 | 389 | 767 | -11 |
| LIONESS02 | 341 | 410 | 751 | -69 |
| LIONESS04 | 231 | 264 | 495 | -33 |
| LIONESS06 | 467 | 443 | 910 | 24 |
| LIONESS09 | 460 | 560 | 1020 | -100 |
| LIONESS10 | 510 | 455 | 965 | 55 |
| LIONESS12 | 432 | 389 | 821 | 43 |
| LIONESS13 | 479 | 616 | 1095 | -137 |
| LIONESS17 | 236 | 360 | 596 | -124 |
| LIONESS20 | 510 | 526 | 1036 | -16 |
| LIONESS23 | 465 | 480 | 945 | -15 |
| LIONESS24 | 220 | 223 | 443 | -3 |
|  |  | **Mean =** | **820.33** | **-32.17** |
|  |  | **SD =** | **215.86** | **62.99** |
| **GROUP B PATIENT** | **ETT2 Lira1.8 Time to max STD (secs)** | **ETT4 Placebo Time to max STD (secs)** | **ETT2 + ETT4** | **ETT2 - ETT4** |
| LIONESS03 | 689 | 711 | 1400 | -22 |
| LIONESS05 | 193 | 216 | 409 | -23 |
| LIONESS07 | 500 | 570 | 1070 | -70 |
| LIONESS08 | 90 | 547 | 637 | -457 |
| LIONESS11 | 636 | 570 | 1206 | 66 |
| LIONESS14 | 587 | 596 | 1183 | -9 |
| LIONESS15 | 557 | 452 | 1009 | 105 |
| LIONESS16 | 512 | 530 | 1042 | -18 |
| LIONESS18 | 261 | 265 | 526 | -4 |
| LIONESS19 | 290 | 240 | 530 | 50 |
|  |  | **Mean =** | **901.20** | **-38.20** |
|  |  | **SD =** | **345.07** | **155.80** |
| Key: ETT=exercise treadmill test; Lira=liraglutide; secs=seconds; SD=standard deviation; STD=ST-segment depression | | | | |

**Supplemental Table 11**

**Change in total exercise time achieved during ETT-1 versus ETT-3**

| **GROUP A PATIENT** | **ETT1 Placebo Total**  **Exercise Time (secs)** | **ETT3 Lira1.2 Total**  **Exercise Time (secs)** | **ETT1 + ETT3** | **ETT1 - ETT3** |
| --- | --- | --- | --- | --- |
| LIONESS01 | 367 | 372 | 739 | -5 |
| LIONESS02 | 306 | 409 | 715 | -103 |
| LIONESS04 | 233 | 223 | 456 | 10 |
| LIONESS06 | 496 | 550 | 1046 | -54 |
| LIONESS09 | 531 | 469 | 1000 | 62 |
| LIONESS10 | 518 | 524 | 1042 | -6 |
| LIONESS12 | 243 | 295 | 538 | -52 |
| LIONESS13 | 554 | 558 | 1112 | -4 |
| LIONESS17 | 199 | 268 | 467 | -69 |
| LIONESS20 | 526 | 465 | 991 | 61 |
| LIONESS23 | 506 | 458 | 964 | 48 |
| LIONESS24 | 240 | 251 | 491 | -11 |
|  |  | **Mean =** | **796.75** | **-10.25** |
|  |  | **SD =** | **256.55** | **52.13** |
| **GROUP B PATIENT** | **ETT1 Lira 1.2 Total**  **Exercise Time (secs)** | **ETT3 Placebo Total**  **Exercise Time (secs)** | **ETT1 + ETT3** | **ETT1 - ETT3** |
| LIONESS03 | 646 | 687 | 1333 | -41 |
| LIONESS05 | 167 | 201 | 368 | -34 |
| LIONESS07 | 459 | 538 | 997 | -79 |
| LIONESS08 | 245 | 531 | 776 | -286 |
| LIONESS11 | 247 | 659 | 906 | -412 |
| LIONESS14 | 523 | 647 | 1170 | -124 |
| LIONESS15 | 505 | 521 | 1026 | -16 |
| LIONESS16 | 402 | 565 | 967 | -163 |
| LIONESS18 | 260 | 313 | 573 | -53 |
| LIONESS19 | 230 | 253 | 483 | -23 |
|  |  | **Mean =** | **859.9** | **-123.1** |
|  |  | **SD =** | **307.92** | **131.07** |
| Key: ETT=exercise treadmill test; Lira=liraglutide; secs=seconds; SD=standard deviation | | | | |

**Supplemental Table 12**

**Change in total exercise time achieved during ETT-2 versus ETT-4**

| **GROUP A PATIENT** | **ETT2 Placebo Total**  **Exercise Time (secs)** | **ETT4 Lira1.8 Total**  **Exercise Time (secs)** | **ETT2 + ETT4** | **ETT2 - ETT4** |
| --- | --- | --- | --- | --- |
| LIONESS01 | 378 | 389 | 767 | -11 |
| LIONESS02 | 341 | 410 | 751 | -69 |
| LIONESS04 | 231 | 264 | 495 | -33 |
| LIONESS06 | 473 | 443 | 916 | 30 |
| LIONESS09 | 559 | 561 | 1120 | -2 |
| LIONESS10 | 519 | 454 | 973 | 65 |
| LIONESS12 | 431 | 398 | 829 | 33 |
| LIONESS13 | 478 | 616 | 1094 | -138 |
| LIONESS17 | 236 | 360 | 596 | -124 |
| LIONESS20 | 546 | 526 | 1072 | 20 |
| LIONESS23 | 510 | 485 | 995 | 25 |
| LIONESS24 | 242 | 251 | 493 | -9 |
|  |  | **Mean =** | **841.75** | **-17.75** |
|  |  | **SD =** | **224.93** | **63.20** |
| **GROUP B PATIENT** | **ETT 2 Lira1.8 Total**  **Exercise Time (secs)** | **ETT 4 Placebo Total**  **Exercise Time (secs)** | **ETT2 + ETT4** | **ETT2 - ETT4** |
| LIONESS03 | 690 | 711 | 1401 | -21 |
| LIONESS05 | 192 | 216 | 408 | -24 |
| LIONESS07 | 499 | 570 | 1069 | -71 |
| LIONESS08 | 275 | 547 | 822 | -272 |
| LIONESS11 | 636 | 598 | 1234 | 38 |
| LIONESS14 | 587 | 596 | 1183 | -9 |
| LIONESS15 | 557 | 452 | 1009 | 105 |
| LIONESS16 | 512 | 554 | 1066 | -42 |
| LIONESS18 | 261 | 264 | 525 | -3 |
| LIONESS19 | 290 | 246 | 536 | 44 |
|  |  | **Mean =** | **925.3** | **-25.5** |
|  |  | **SD =** | **337.56** | **99.89** |
| Key: ETT=exercise treadmill test; Lira=liraglutide; secs=seconds; SD=standard deviation | | | | |

**Supplemental Table 13**

**Change in recovery time to 0.05 mV ST-segment depression during ETT-1 versus ETT-3**

| **GROUP A PATIENT** | **ETT1 Placebo Recovery time (secs)** | **ETT3 Lira1.2 Recovery time (secs)** | **ETT1 + ETT3** | **ETT1 - ETT3** |
| --- | --- | --- | --- | --- |
| LIONESS01 | 530 | 475 | 1005 | 55 |
| LIONESS02 | 380 | 374 | 754 | 6 |
| LIONESS04 | 60 | 107 | 167 | -47 |
| LIONESS06 | 85 | 20 | 105 | 65 |
| LIONESS09 | 40 | 19 | 59 | 21 |
| LIONESS10 | 60 | 39 | 99 | 21 |
| LIONESS12 | 59 | 70 | 129 | -11 |
| LIONESS13 | 141 | 68 | 209 | 73 |
| LIONESS17 | 41 | 76 | 117 | -35 |
| LIONESS20 | 520 | 320 | 840 | 200 |
| LIONESS23 | 520 | 370 | 890 | 150 |
| LIONESS24 | 60 | 60 | 120 | 0 |
|  |  | **Mean =** | **374.50** | **41.50** |
|  |  | **SD =** | **373.42** | **73.17** |
| **GROUP B PATIENT** | **ETT1 Lira1.2 Recovery time (secs)** | **ETT3 Placebo Recovery time (secs)** | **ETT1 + ETT3** | **ETT1 - ETT3** |
| LIONESS03 | 523 | 500 | 1023 | 23 |
| LIONESS05 | 381 | 433 | 814 | -52 |
| LIONESS07 | 379 | 175 | 554 | 204 |
| LIONESS08 | 360 | 362 | 722 | -2 |
| LIONESS11 | 27 | 53 | 80 | -26 |
| LIONESS14 | 350 | 500 | 850 | -150 |
| LIONESS15 | 51 | 40 | 91 | 11 |
| LIONESS16 | 60 | 106 | 166 | -46 |
| LIONESS18 | 294 | 70 | 364 | 224 |
| LIONESS19 | 74 | 120 | 194 | -46 |
|  |  | **Mean =** | **485.80** | **14.00** |
|  |  | **SD =** | **351.53** | **115.76** |
| Key: ETT=exercise treadmill test; Lira=liraglutide; secs=seconds; SD=standard deviation | | | | |

**Supplemental Table 14**

**Change in recovery time to 0.05 mV ST-segment depression during ETT-2 versus ETT-4**

| **GROUP A PATIENT** | **ETT2 Placebo Recovery time (secs)** | **ETT4 Lira1.8 Recovery time (secs)** | **ETT2 + ETT4** | **ETT2 - ETT4** |
| --- | --- | --- | --- | --- |
| LIONESS01 | 470 | 410 | 880 | 60 |
| LIONESS02 | 300 | 433 | 733 | -133 |
| LIONESS04 | 61 | 60 | 121 | 1 |
| LIONESS06 | 61 | 40 | 101 | 21 |
| LIONESS09 | 40 | 40 | 80 | 0 |
| LIONESS10 | 55 | 40 | 95 | 15 |
| LIONESS12 | 80 | 50 | 130 | 30 |
| LIONESS13 | 60 | 40 | 100 | 20 |
| LIONESS17 | 91 | 171 | 262 | -80 |
| LIONESS20 | 33 | 340 | 373 | -307 |
| LIONESS23 | 490 | 405 | 895 | 85 |
| LIONESS24 | 42 | 40 | 82 | 2 |
|  |  | **Mean =** | **321.00** | **-23.83** |
|  |  | **SD =** | **324.44** | **106.30** |
| **GROUP B PATIENT** | **ETT2 Lira1.8 Recovery time (secs)** | **ETT4 Placebo Recovery time (secs)** | **ETT2 + ETT4** | **ETT2 - ETT4** |
| LIONESS03 | 420 | 540 | 960 | -120 |
| LIONESS05 | 180 | 120 | 300 | 60 |
| LIONESS07 | 340 | 510 | 850 | -170 |
| LIONESS08 | 15 | 0 | 15 | 15 |
| LIONESS11 | 14 | 112 | 126 | -98 |
| LIONESS14 | 458 | 594 | 1052 | -136 |
| LIONESS15 | 70 | 80 | 150 | -10 |
| LIONESS16 | 100 | 136 | 236 | -36 |
| LIONESS18 | 117 | 105 | 222 | 12 |
| LIONESS19 | 110 | 84 | 194 | 26 |
|  |  | **Mean =** | **410.50** | **-45.70** |
|  |  | **SD =** | **385.42** | **79.27** |
| Key: ETT=exercise treadmill test; Lira=liraglutide; secs=seconds; SD=standard deviation | | | | |

**Supplemental Table 15**

**Frequency of angina episodes during the LIONESS trial**

| **Group A** | WEEK 1 | WEEK 2 | WEEK 3 | **Mean** | WEEK 4 | WEEK 5 | WEEK 6 | **Mean** |
| --- | --- | --- | --- | --- | --- | --- | --- | --- |
| LIONESS01 | 2 | 0 | 0 | **1** | 1 | 2 | 1 | **1** |
| LIONESS02 | 0 | 0 | 1 | **0** | 0 | 0 | 0 | **0** |
| LIONESS04 | 1 | 2 | 1 | **1** | 1 | 1 | 2 | **1** |
| LIONESS06 | 2 | 0 | 0 | **1** | 0 | 0 | 0 | **0** |
| LIONESS09 | 0 | 0 | 0 | **0** | 1 | 2 | 0 | **1** |
| LIONESS10 | 0 | 0 | 0 | **0** | 0 | 0 | 0 | **0** |
| LIONESS12 | 5 | 4 | 2 | **4** | 0 | 0 | 0 | **0** |
| LIONESS13 | 1 | 1 | 0 | **1** | 1 | 0 | 2 | **1** |
| LIONESS17 | 1 | 0 | 0 | **0** | 1 | 0 | 0 | **0** |
| LIONESS20 | 0 | 0 | 0 | **0** | 0 | 1 | 1 | **1** |
| LIONESS23 | 0 | 0 | 0 | **0** | 0 | 0 | 0 | **0** |
| LIONESS24 | 0 | 2 | 0 | **1** | 0 | 0 | 0 | **0** |
| **Group B** | WEEK 1 | WEEK 2 | WEEK 3 | **Mean** | WEEK 4 | WEEK 5 | WEEK 6 | **Mean** |
| LIONESS03 | 0 | 0 | 1 | **0** | 2 | 2 | 0 | **1** |
| LIONESS05 | 1 | 0 | 1 | **1** | 0 | 0 | 0 | **0** |
| LIONESS07 | 1 | 0 | 0 | **0** | 0 | 0 | 0 | **0** |
| LIONESS08 | 0 | 0 | 0 | **0** | 0 | 0 | 2 | **1** |
| LIONESS11 | 2 | 2 | 1 | **2** | 0 | 0 | 1 | **0** |
| LIONESS14 | 1 | 0 | 0 | **0** | 0 | 0 | 0 | **0** |
| LIONESS15 | 0 | 0 | 0 | **0** | 1 | 2 | 1 | **1** |
| LIONESS16 | 0 | 0 | 0 | **0** | 1 | 0 | 2 | **1** |
| LIONESS18 | 4 | 0 | 3 | **2** | 9 | 3 | 5 | **6** |
| LIONESS19 | 3 | 1 | 0 | **1** | 7 | 2 | 5 | **5** |

**Supplemental Table 16**

**Frequency of gastrointestinal episodes during the LIONESS trial**

| **Group A** | WEEK 1 | WEEK 2 | WEEK 3 | **Mean** | WEEK 4 | WEEK 5 | WEEK 6 | **Mean** |
| --- | --- | --- | --- | --- | --- | --- | --- | --- |
| LIONESS01 | 0 | 0 | 0 | **0** | 0 | 0 | 0 | **0** |
| LIONESS02 | 0 | 0 | 0 | **0** | 0 | 0 | 0 | **0** |
| LIONESS04 | 0 | 0 | 0 | **0** | 0 | 0 | 2 | **1** |
| LIONESS06 | 0 | 0 | 0 | **0** | 0 | 0 | 0 | **0** |
| LIONESS09 | 2 | 2 | 0 | **1** | 0 | 5 | 0 | **2** |
| LIONESS10 | 0 | 0 | 0 | **0** | 8 | 0 | 0 | **3** |
| LIONESS12 | 0 | 0 | 0 | **0** | 0 | 0 | 0 | **0** |
| LIONESS13 | 0 | 0 | 1 | **0** | 1 | 3 | 0 | **1** |
| LIONESS17 | 0 | 0 | 0 | **0** | 0 | 0 | 0 | **0** |
| LIONESS20 | 0 | 0 | 0 | **0** | 0 | 0 | 0 | **0** |
| LIONESS23 | 0 | 0 | 0 | **0** | 0 | 0 | 0 | **0** |
| LIONESS24 | 0 | 0 | 0 | **0** | 0 | 0 | 0 | **0** |
| **Group B** | WEEK 1 | WEEK 2 | WEEK 3 | **Mean** | WEEK 4 | WEEK 5 | WEEK 6 | **Mean** |
| LIONESS03 | 1 | 0 | 0 | **0** | 0 | 0 | 0 | **0** |
| LIONESS05 | 1 | 1 | 1 | **1** | 0 | 0 | 0 | **0** |
| LIONESS07 | 0 | 0 | 0 | **0** | 0 | 0 | 0 | **0** |
| LIONESS08 | 0 | 0 | 1 | **0** | 0 | 0 | 0 | **0** |
| LIONESS11 | 1 | 4 | 0 | **2** | 0 | 0 | 0 | **0** |
| LIONESS14 | 2 | 0 | 0 | **1** | 0 | 0 | 0 | **0** |
| LIONESS15 | 0 | 1 | 0 | **0** | 0 | 0 | 0 | **0** |
| LIONESS16 | 0 | 0 | 1 | **0** | 0 | 0 | 0 | **0** |
| LIONESS18 | 0 | 2 | 0 | **1** | 0 | 0 | 0 | **0** |
| LIONESS19 | 0 | 0 | 0 | **0** | 0 | 0 | 0 | **0** |

**Supplemental Figure 1**

**Home blood glucose monitoring during the LIONESS trial**

Plate A: Average blood glucose levels in the morning for all participants

Plate B: Average blood glucose levels in the afternoon for all participants


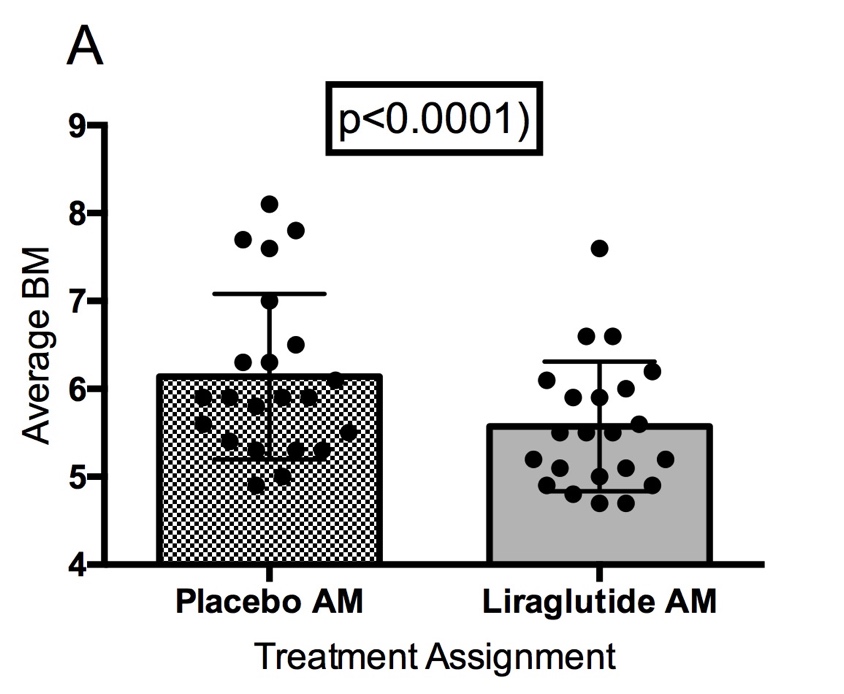


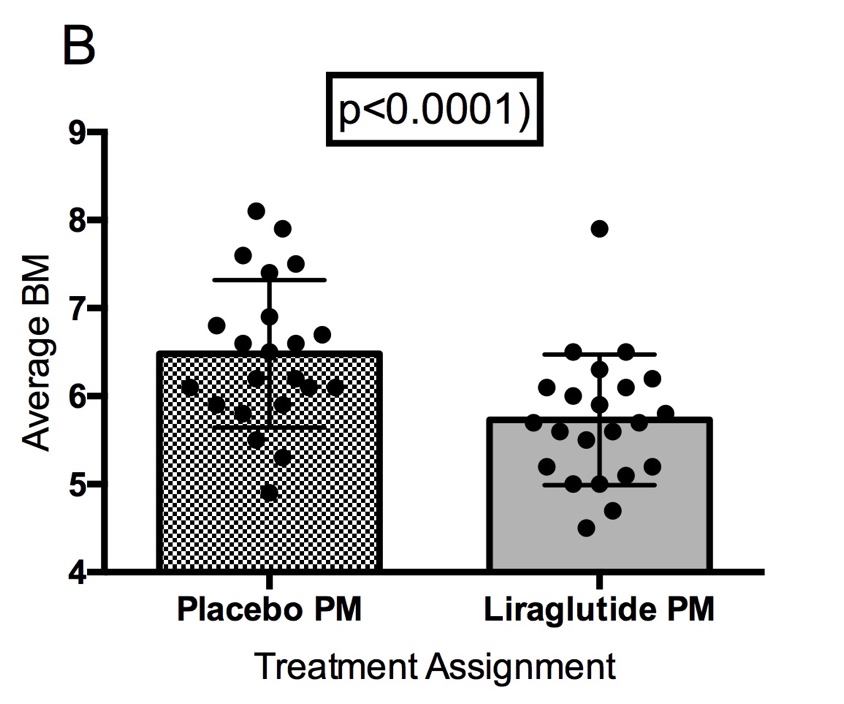


**Supplemental Figure 2**

**Random plasma glucose multiple comparisons during the LIONESS trial**

**
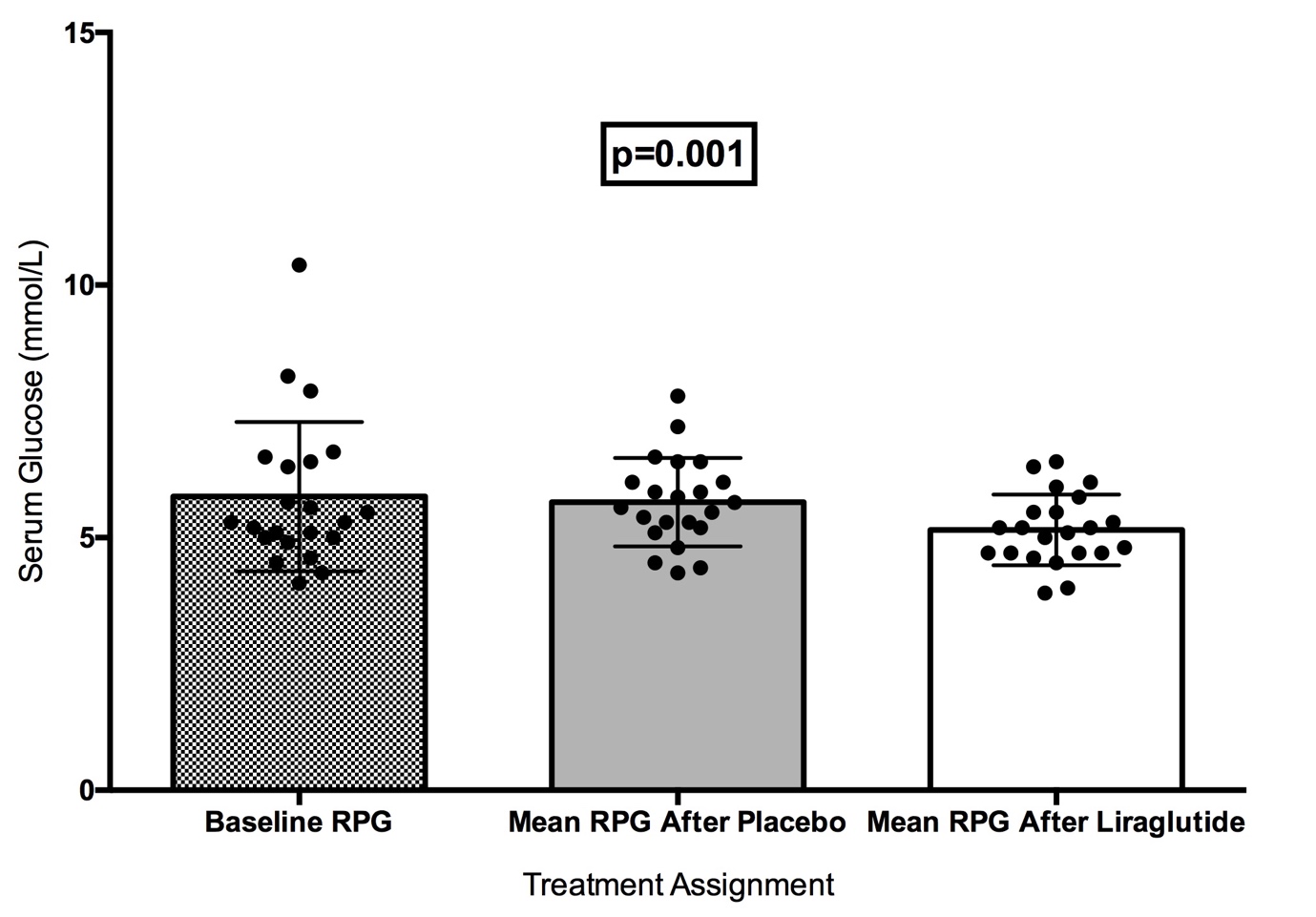
**

**Supplemental Figure 3**

**Assessment of renal function during the LIONESS trial**

**Plate A**: Mean serum creatinine

**Plate B**: Mean estimated glomerular filtration rate (eGFR)


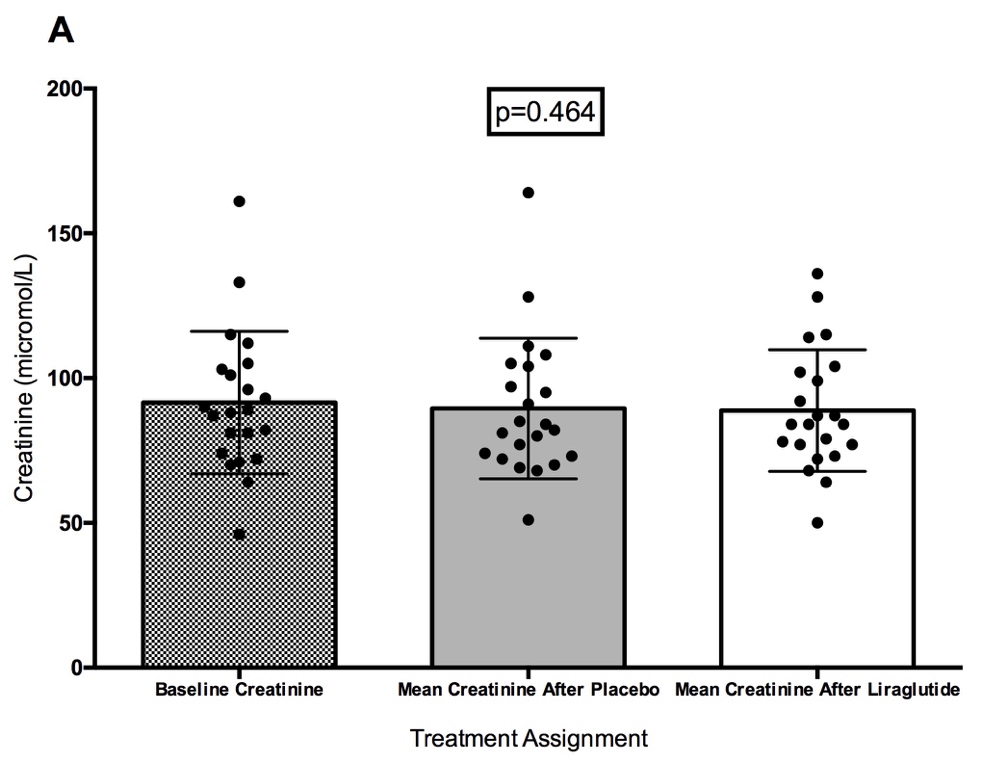


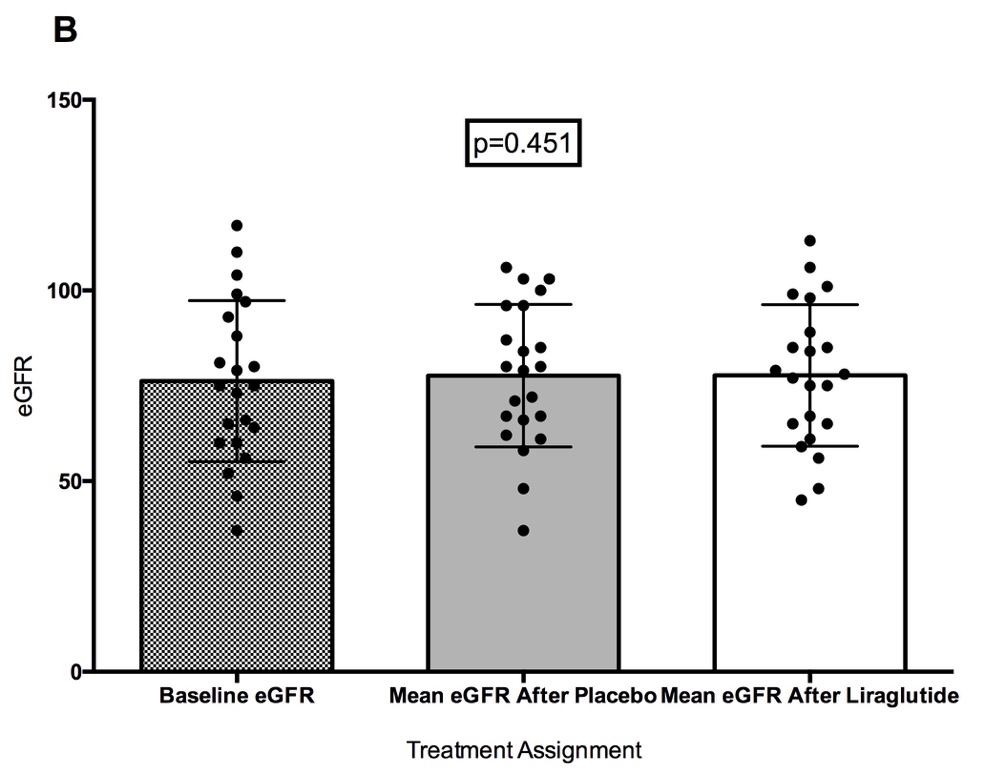


**Supplemental Figure 4**

**Serum amylase multiple comparisons for the assessment of acute pancreatitis**

**
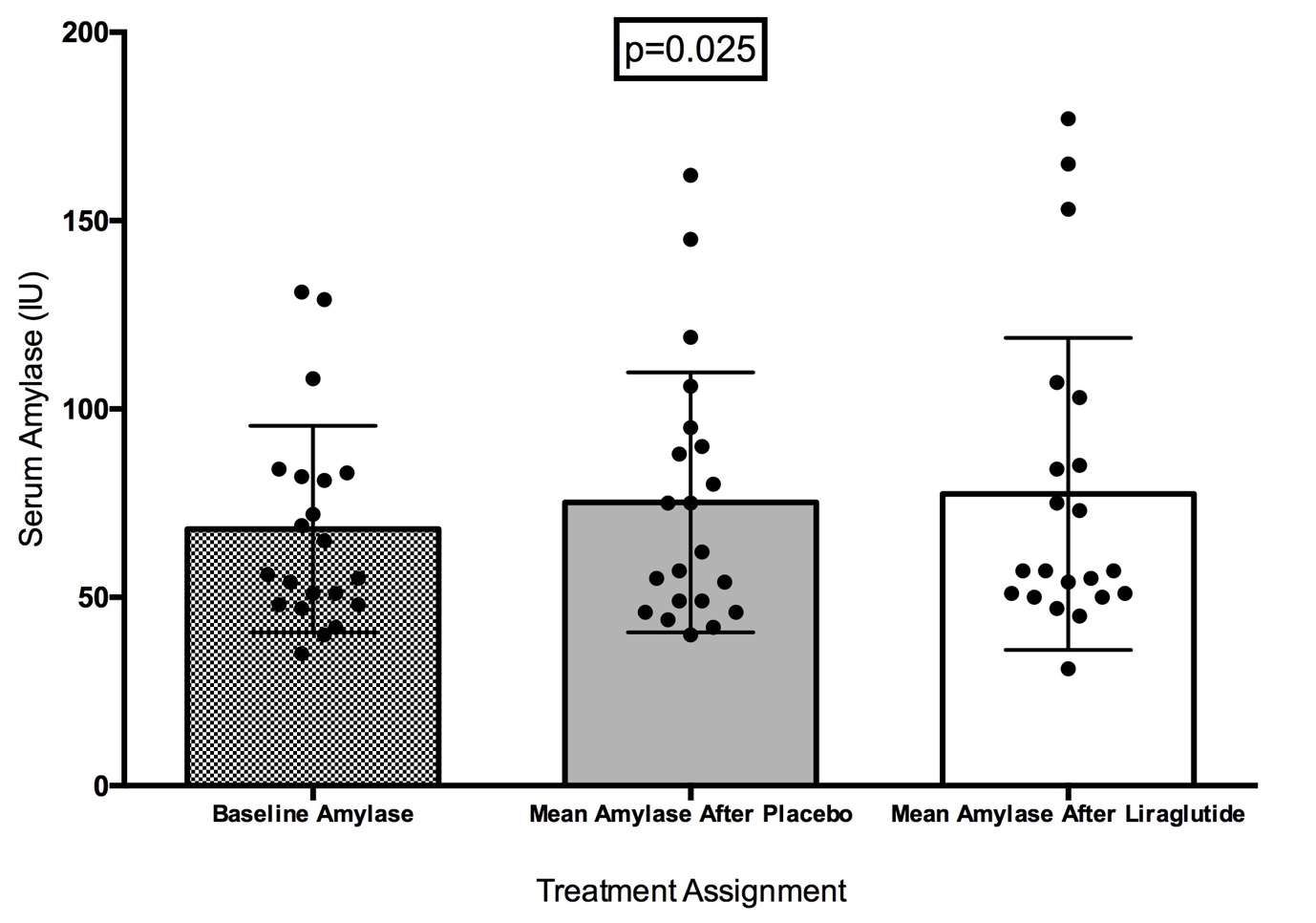
**

**Supplemental Figure 5**

**Multiple comparisons of mean weight at baseline and after each treatment period of the trial protocol**

**
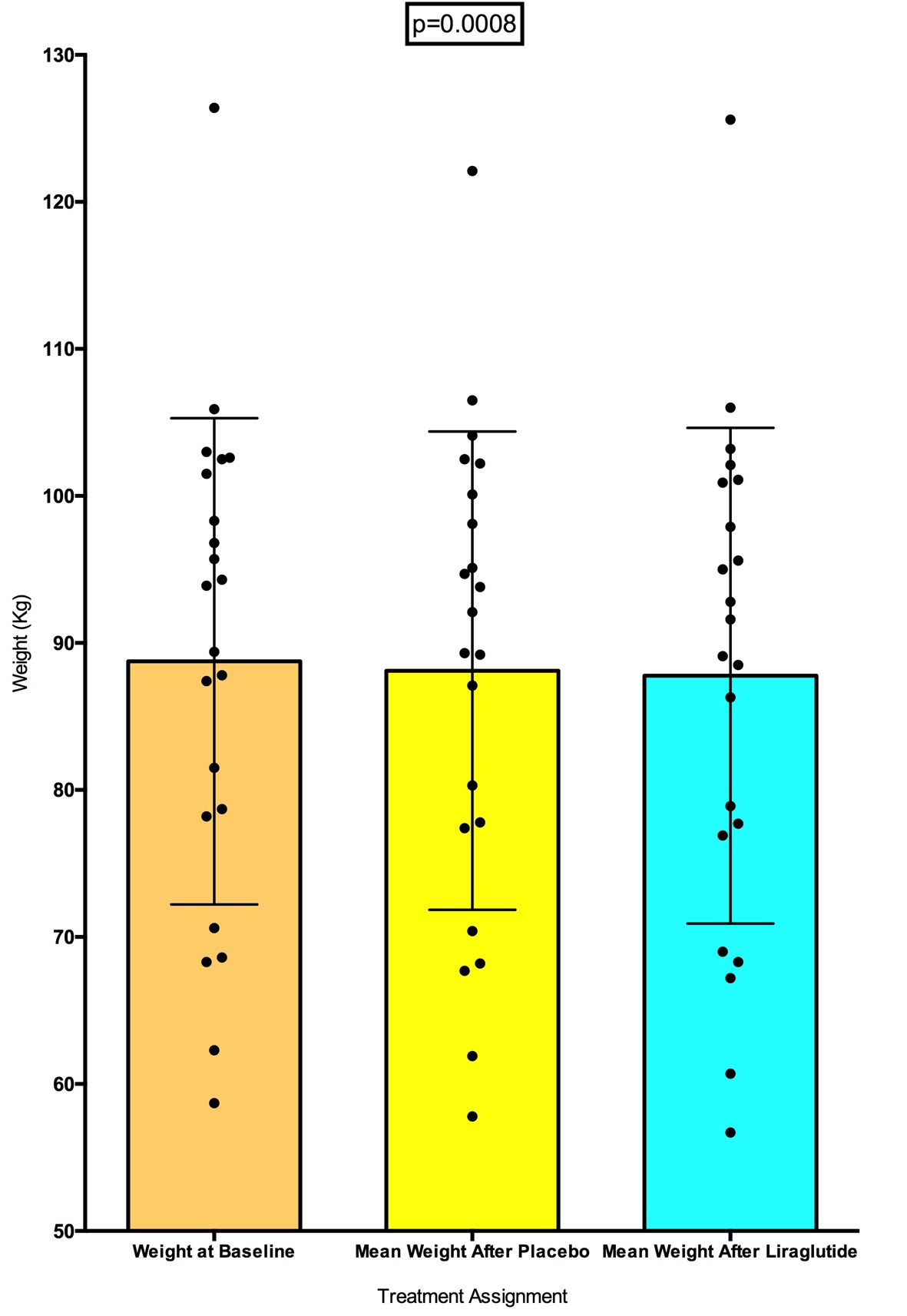
**

**Supplemental Figure 6**

**Multiple comparisons of blood pressure at baseline and after trial completion**

**Plate A**: Mean systolic blood pressure

**Plate B**: Mean diastolic blood pressure


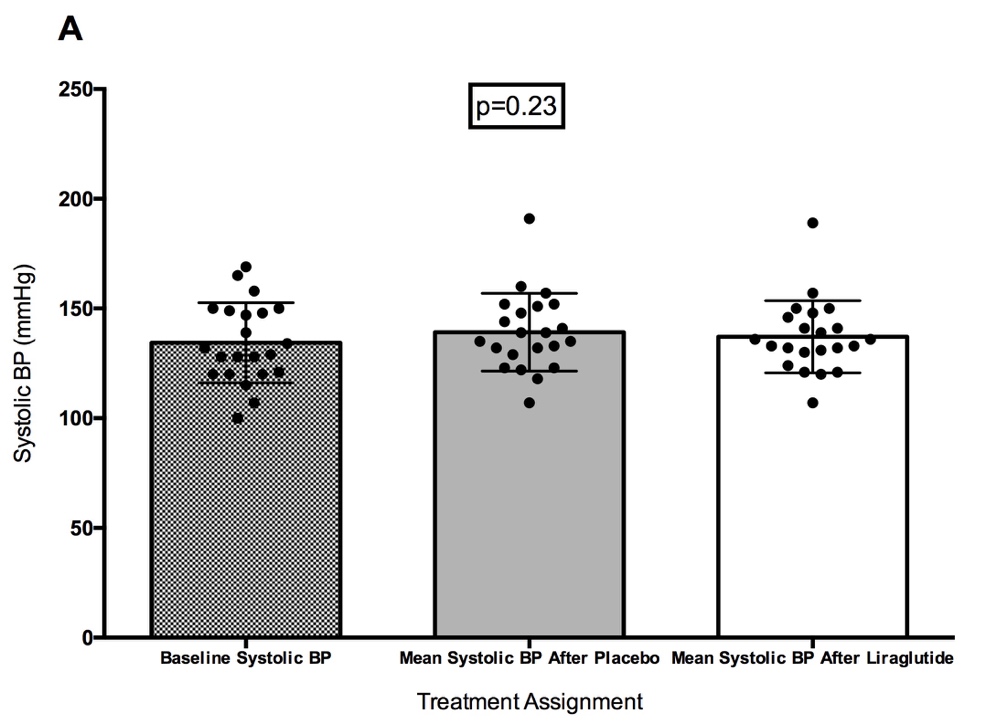


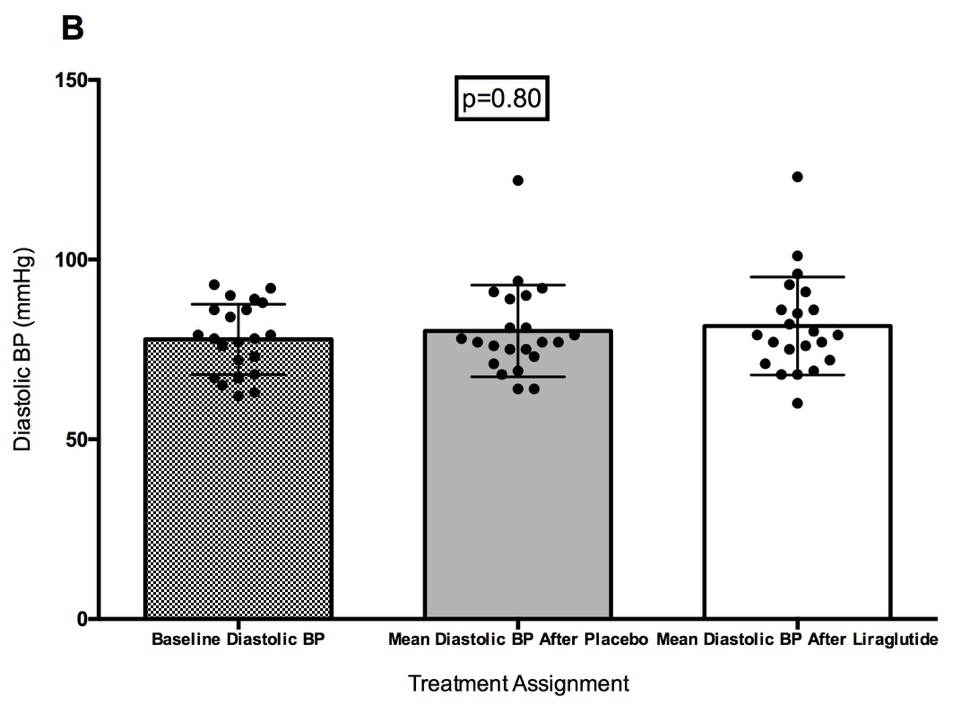


**Supplemental Figure 7**

**Assessment of symptoms over the course of 6 weeks**

**Plate A**: Mean angina episodes

**Plate B**: Mean gastrointestinal side effects


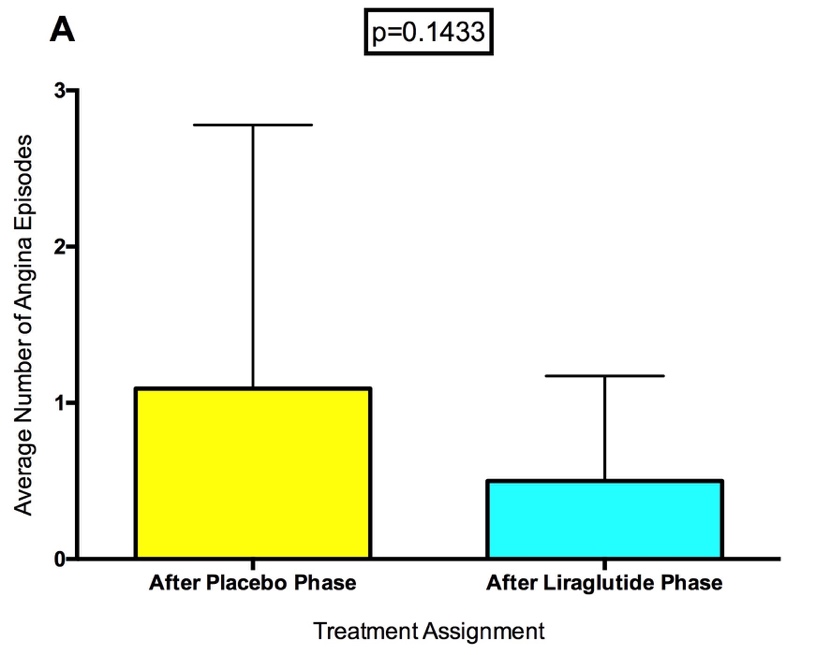


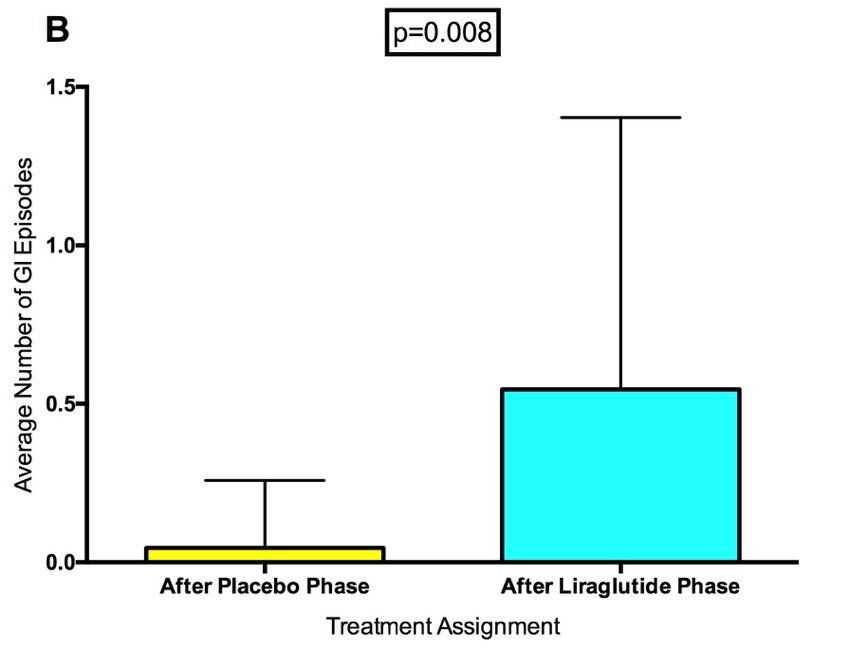

Supplement: Supplementary file 1 — Additional file 1: LIONESS Trial Supplementary Material [file 13098_2021_635_MOESM1_ESM.docx]
